# Digital Twin-Enabled Vehicle Routing in Logistics and Transportation

**DOI:** 10.3390/s26195986

**Published:** 2026-09-22

**Authors:** Amir Alavi, Hamidreza Alavi

**Affiliations:** 1 Faculty of Commerce and Business Administration, Department of Business, Douglas College, 700 Royal Ave, New Westminster, BC V3M 5Z5, Canada; amiralavi13@gmail.com; 2 Department of Engineering, University of Cambridge, Cambridge CB3 0FA, UK; 3 School of the Built Environment, Oxford Brookes University, Oxford OX3 0BP, UK

**Keywords:** digital twin, vehicle routing problem, intelligent transportation systems, Internet of Vehicles, cyber–physical systems, real-time sensing, dynamic routing, logistics, PRISMA, decision support

## Abstract

Vehicle routing in modern logistics increasingly depends on live sensing: GPS and Internet-of-Things telemetry, roadside perception, and vehicle-to-everything (V2X) data streams change the operational state faster than static route plans can absorb. Digital twins have been proposed to link real-time sensing with routing decisions, yet the literature is fragmented across production, emergency, urban, supply-chain, and Internet of Vehicles (IoV) domains. This systematic review synthesizes 30 peer-reviewed studies of digital-twin-enabled vehicle routing retrieved by a two-stage search of Scopus, ScienceDirect, IEEE Xplore, and Google Scholar, and reported in accordance with PRISMA 2020. Each study was coded for domain, digital-twin role, coupling granularity, evidence maturity, evidence type, and evidence confidence. Four roles recur in the routing loop: monitoring and synchronization; simulation and training; adaptive re-optimization; and infrastructure, data governance, and model management. Twenty-five studies contribute primary evidence and five are conceptual or secondary review sources; simulation-only validation dominates (19 of 30 studies), and only three studies are validated on industrial or field data, so reported operational benefits remain largely unconfirmed. No meta-analysis was performed, because the corpus is too heterogeneous for pooled estimation. The review contributes a role taxonomy, an evidence-maturity mapping, and a routing-specific reference architecture in which synchronization quality, decision latency, and sensor-data governance are proposed as first-class design variables, together with three testable design hypotheses and a research agenda for sensing-driven, cyber-physical routing systems.

## 1. Introduction

Logistics and transportation systems operate under dynamic and uncertain conditions driven by fluctuating customer demand, congestion in urban networks, volatile production schedules, and time-critical emergency operations [1,2]. Efficient operation requires balancing service level, transportation cost, and inventory holding, yet this balance becomes progressively harder to maintain as networks grow in scale, connectivity, and data intensity. High mobility, heterogeneous sensor and telemetry sources, and tightening sustainability expectations mean that routing decisions can no longer be treated as periodic planning exercises; they must respond continuously to conditions that change faster than conventional planning cycles can absorb.

For several decades, the vehicle routing problem (VRP) has provided the foundational model for such decisions, determining cost-efficient routes for a fleet subject to capacity, time-window, distance, and precedence constraints [3,4,5]. Variants including the VRP with pick-up and delivery (VRPPD), the dynamic VRP (DVRP), time-dependent formulations, and multi-hop routing in connected networks have extended this model to more demanding settings [6,7]. Classical formulations nevertheless rely largely on static or periodically updated inputs and struggle to respond proactively to real-time disruptions, stochastic travel conditions, and rapidly evolving requirements, which manifests as delay, energy waste, and degraded service [8,9].

Static VRP formulations struggle under five conditions that recur in modern logistics: (i) the system state changes during execution, so a plan computed at dispatch is quickly outdated; (ii) travel times are stochastic rather than fixed; (iii) constraints emerge after dispatch, such as new orders, incidents, or vehicle faults; (iv) the communication or network state affects physical routing, as in connected and autonomous fleets; and (v) energy and charging constraints interact dynamically with route choice, as in electric freight. These are precisely the situations in which a static or periodically updated plan loses validity.

Digital twin (DT) technology offers a complementary paradigm. A DT is a virtual, continuously updated representation of a physical system that synchronizes state, behavior, and context through real-time sensing and computational modeling [10,11,12]. In logistics and transportation, the synchronization is fed by onboard telematics, roadside perception, IoT tags, and V2X messaging, so the twin is in effect the point at which heterogeneous sensor streams are fused into a single decision-relevant state estimate. DTs can mirror vehicles, infrastructure, and operating environments; provide safe simulation environments for learning and scenario testing; and support adaptive, data-driven decision-making. When coupled with VRP models, a DT can convert routing from a reactive activity into a closed-loop cyber-physical process that anticipates disruption, evaluates alternatives, and re-optimizes in near real time [6,13,14].

Relative to a static optimizer, a digital twin contributes five capabilities: (i) live synchronization of system state from streaming sensor data; (ii) simulation of candidate actions before they are taken; (iii) closed-loop re-optimization as conditions change; (iv) integration of cyber and physical routing within a single representation; and (v) a governance and model-management layer that makes deployment at scale tractable. These capabilities, rather than any single algorithm, distinguish DT-enabled routing from conventional dynamic VRP and motivate the cross-domain synthesis that follows.

Research at this intersection has expanded rapidly but unevenly. DT-enabled routing has been demonstrated for production and intralogistics, including automated container terminals [6,13,15,16]; emergency and healthcare logistics [9,14]; urban mobility and smart transportation, including traffic-aware electric-vehicle routing and charging [8,17,18]; supply-chain, warehousing, cold-chain, and energy-aware logistics [19,20,21,22]; and Internet of Vehicles (IoV) communication routing and DT infrastructure [7,23,24]. These literatures employ different vocabularies, evaluation practices, and architectural assumptions, and they rarely reference one another, leaving the field fragmented and hard to compare across contexts.

Existing reviews of DTs in logistics or intelligent transportation tend to treat physical-goods routing and cyber/communication routing separately, and to remain at the level of transport planning or general DT management rather than the routing decision itself [25,26]. To the authors’ knowledge, no prior review systematically synthesizes DT-enabled routing across both physical logistics and cyber/IoV routing while explicitly coding the role of the twin, its coupling granularity, and the maturity of the supporting evidence. This gap matters because the contribution of a DT—and the conditions under which it improves routing—differ fundamentally across these roles, and conflating them obscures where the field is strong, where it is mainly conceptual, and where the evidence remains limited.

This paper addresses that gap through a systematic review of 30 studies, reported in accordance with PRISMA 2020 [27]. Its objectives are to: (1) classify and compare DT-enabled routing across five application domains; (2) develop an analytical synthesis using cross-cutting lenses—DT role, coupling granularity, evidence maturity, solver class, and evaluation metrics; (3) consolidate the evidence into a reference architecture for DT-enabled vehicle routing, framed not as a novel block diagram but as an evidence-derived structure with testable design hypotheses; and (4) articulate a research agenda linking identified gaps to the underlying evidence. Throughout, the review distinguishes decisions about the movement of vehicles and goods—its primary object—from cyber-layer routing decisions about the movement of information and computation, which are included only where they condition the routing decision loop (Section 3.3). The remainder of the paper is organized as follows. Section 2 provides background on VRP and DT concepts; Section 3 details the review methodology; Section 4 gives a descriptive overview of the corpus; Section 5 presents the thematic classification; Section 6 develops the cross-theme analytical synthesis; Section 7 presents the reference architecture; Section 8 discusses major debates; and Section 9, Section 10 and Section 11 set out the research agenda, limitations, and conclusions.

## 2. Background

### 2.1. Vehicle Routing Problems and Dynamic Routing

The VRP originates with the truck-dispatching formulation of Dantzig and Ramser [3] and has become one of the most studied problems in operations research, supported by mature exact and approximate solution methods [4,5]. In its classical form, the VRP seeks minimum-cost routes for a fleet serving geographically dispersed customers subject to capacity, time-window, distance, or precedence constraints. Successive variants—VRPPD, DVRP, time-dependent and stochastic VRPs, and multi-hop routing in communication networks—have progressively relaxed the assumption of a static, fully known environment [6,7].

Dynamic and stochastic routing acknowledge that information arrives over time and that plans must be revised during execution. In high-mobility settings such as smart cities, industrial parks, and emergency response, conditions such as congestion, incidents, equipment failure, and demand surges evolve continuously, so a route that is optimal at dispatch can be obsolete on deployment [8,14]. The central limitation of conventional approaches is therefore not solver quality but the freshness and fidelity of the state on which the solver operates: without a continuously updated representation of the physical system, even sophisticated optimizers act on stale inputs.

### 2.2. Digital Twin Concepts in Logistics and Transportation

The digital twin concept, articulated by Grieves and Vickers [10] and consolidated in subsequent surveys [11,12], describes a coupled physical–virtual system in which a virtual model is kept synchronized with a physical asset through bidirectional data flows, commonly comprising physical entities, virtual models, data, services, and their connections. A DT is distinguished from a conventional simulation by this live coupling: it is updated by streaming sensor data and can act back on the physical system. Kritzinger et al. [28] sharpen this distinction into three levels that this review adopts as an eligibility instrument in Section 3.3: a digital modelwith no automated data exchange; a digital shadow with automated one-way flow from the physical system to the model; and a digital twin proper, in which the flow is bidirectional and the model acts back on the physical system. The quality of a logistics twin is consequently bounded by the sensing infrastructure beneath it—the coverage, accuracy, latency, and reliability of the telemetry that keeps the virtual state aligned with reality.

In logistics and transportation, DTs have been applied to physical infrastructure (warehouses, road networks, ports), mobile assets (trucks, automated guided vehicles (AGVs), ambulances, connected and autonomous vehicles), and broader operational systems (supply-chain processes, traffic ecosystems) [2,19,26]. Recent reviews map this growth at the level of transport planning and document a rapid post-2021 expansion [25], while also observing that many logistics and transport DTs remain at proof-of-concept stage with limited standardization and interoperability [12,20]—observations this review tests against the routing-specific evidence.

Beyond transport and logistics, DT research in the built environment has matured in parallel. That literature is used here for one purpose only: to identify transferable design concepts. It is not treated as evidence for DT-enabled vehicle routing, it was never a candidate for the review corpus, and none of it is cited in support of any claim about routing performance. Five elements transfer. The first is asset-state representation: BIM-based decision-support systems maintain an explicit, semantically structured model of the asset that a decision can be posed against [29,30,31]. The second is physical–virtual synchronization: integrating BIM with LiDAR point clouds to keep a model aligned with the as-is asset [32] is the built-environment counterpart of the state-alignment problem that is central to routing twins. The third is model updating, the procedure by which a divergence between model and reality is detected and corrected. The fourth is sensor-data governance, including ownership, quality, and access control over the streams that keep the model current. The fifth is operational decision support, the discipline of specifying which decision the model exists to serve [30]. A review of DT applications for building energy efficiency [33] additionally offers a methodological precedent for stocktaking a fragmented DT literature. What this body of work does not provide is any evidence about routing, dispatching, or mobility decisions; its relevance is confined to the design concepts listed above, which become material once routing decisions are embedded in sensed cyber-physical infrastructure.

### 2.3. Why DT–VRP Integration Matters

Coupling a DT with a VRP targets precisely the limitation identified in Section 2.1: it supplies the optimizer with a continuously synchronized, predictive representation of the operating environment, enabling anticipation rather than reaction [6,9]. Three mechanisms recur. First, synchronization keeps the routing state current, so decisions reflect live positions, congestion, and disruptions. Second, the twin serves as a safe environment in which learning agents are trained and what-if scenarios evaluated before deployment [13,16]. Third, the twin closes the loop by updating parameters and re-optimizing as conditions change [18,21].

A further reason the integration matters is the convergence of physical and cyber routing. In connected and autonomous mobility, the quality of physical routing increasingly depends on the reliability of information flow, and DTs are now applied to communication routing, computation offloading, wireless-channel modeling, and the migration of twin models across distributed infrastructure [23,24,34]. Treating these cyber-layer capabilities as conditions on the routing problem, rather than as a separate networking concern, is central to the synthesis developed in this review; the two are not treated as equivalent, and the boundary between them is specified in Section 3.3.

## 3. Review Methodology

### 3.1. Review Protocol and Reporting Framework

The review procedures are the search, eligibility, screening, appraisal, extraction, and synthesis protocol set out in Section 3.2, Section 3.3, Section 3.4, Section 3.5, Section 3.6, Section 3.7 and Section 3.8. PRISMA 2020 [27] was used as a reporting guideline to document that protocol and the flow of records; it was not treated as a methodological framework for generating the procedures themselves. PRISMA-S [35] informed search reporting; the available search-strategy information is summarized in Table 1, and limitations of the search record are disclosed in Section 10. The review protocol was not prospectively registered; the search strategy, screening logic, eligibility criteria, PRISMA flow, appraisal rules, extraction template, and study-level coding matrix are reported instead, so the procedure is auditable. PROSPERO is not an appropriate venue for an engineering and operations-research review; future updates should deposit the protocol in an open repository before screening.

Established clinical risk-of-bias instruments were deliberately not applied: RoB 2 and ROBINS-I address intervention studies and ROBIS appraises reviews rather than primary engineering studies, so none transfers to simulation-based DT–routing research. The Mixed Methods Appraisal Tool informed the appraisal of the few empirical studies; otherwise the tailored appraisal of Section 3.6 was used.

### 3.2. Search Strategy and Information Sources

Searches covered four sources chosen to balance operations-research, engineering, and computer-science venues: Scopus, ScienceDirect (Elsevier), IEEE Xplore, and Google Scholar. They are not equivalent in coverage or reproducibility, and Table 1 summarizes the search strategy. Table 2 states the role and the limitation of each. Two keyword blocks—a digital-twin block and a routing/optimization block—were combined with Boolean operators, deliberately spanning both physical-goods routing (fleets, AGVs) and the cyber routing that conditions DT-driven mobility (DT wireless channels, DT migration, multi-hop DT network routing). The documented strategy is summarized in Table 1, and Figure 1 gives the identification and screening counts.

Two constraints qualify this. The date on which each source was last searched was not recorded and has not been reconstructed. And because Google Scholar ranks results by an undisclosed algorithm and exports no search history, it was used for supplementary discovery and citation chasing rather than as a primary source.

### 3.3. Inclusion and Exclusion Criteria

Studies were assessed against pre-specified eligibility criteria (Table 3) designed to retain work in which a digital twin plays an operational role in a routing, path-planning, dispatching, charging-route, or multi-hop communication-routing decision, and to exclude work that uses routing without a twin or invokes a twin only rhetorically. The same criteria were applied to records from both search stages and to records identified through citation searching.

Two of these criteria carry most of the weight. The first is the substance of the digital twin. Because the term is used loosely in this literature, the Kritzinger et al. [28] ladder was applied as an operational filter, admitting a digital shadow only where its one-way updates informed a routing decision and a digital twin directly, but never a digital model or a data platform on its own (Table 3). The level is recorded per study in Supplementary Materials Table S1: 18 of the 30 studies operationalize a twin in the full sense, 7 sit at the shadow level, and 5 present no operational coupling of their own. Because the corpus is mixed in this respect, the review avoids attributing gains to “the digital twin” as an undifferentiated construct.

The second is the boundary between physical and cyber-layer routing. The primary object of the review is a DT-enabled decision about the movement of vehicles, goods, fleets, responders, or urban traffic. Cyber-layer studies—multi-hop packet routing, offloading, wireless-channel twins, DT migration—were retained only where they affect the availability, latency, reliability, or synchronization of the information such a decision depends on: not because packet routing is the same problem as vehicle routing, but because in IoV-enabled systems, the location, timeliness, and reliability of the twin can constrain the feasible routing decision loop. They are coded separately and read as enabling-infrastructure evidence, never as evidence about physical routing performance. Of the 30 studies, 20 concern a physical routing decision, 3 a cyber-layer routing decision, and 7 supply enabling infrastructure without making a routing decision.

Applied together these criteria are restrictive, which is why a corpus drawn from three very large fields contains only 30 studies: rhetorical DT mentions, generic simulations, pure VRP work without a twin, and DT papers with no routing decision were all excluded, and those categories account for most of what a keyword search on “digital twin” and “routing” returns.

### 3.4. Two-Stage Search and Documented Refinement

Because DT-enabled routing is a fast-moving topic, the search was run in two stages. As the protocol was not prospectively registered, Stage 2 is reported as a documented iterative refinement and update search rather than as a pre-specified amendment. Stage 1 (2019–2025) captured the core DT–routing corpus. Stage 2 (2026) added studies appearing after the initial search and refined the terminology on the basis of what Stage 1 had revealed: (i) author keywords in Stage 1 records, (ii) backward and forward citation checking of Stage 1 studies, (iii) terminology in adjacent DT-routing areas such as intralogistics, cold-chain routing, electric-freight charging, platooning, and DT migration, and (iv) the need to cover the physical–cyber routing interface consistently. The same eligibility criteria, screening procedure, and appraisal were applied to all Stage 2 and citation-search records, and non-peer-reviewed sources were not counted in the 30-study corpus.

Stage 2 contributed 16 of the 30 studies and Stage 1 the other 14; Supplementary Materials Table S1 compares the sub-corpora on every coded dimension. Domain coverage broadened, most visibly in urban mobility (1 study before Stage 2, 7 after) and supply-chain and energy-aware logistics (3 before, 8 after), while emergency and healthcare logistics gained nothing. The DT-role distribution kept its shape. Evidence maturity is the one distribution Stage 2 changed qualitatively: Stage 1 alone holds no prototype- or field-validated study, whereas all 6 entered through Stage 2. That is consistent with a shift toward stronger validation, but Stage 2 also widened the terminology, so the two cannot be separated here and Section 4 draws no inference of methodological maturation. The review’s three central conclusions hold in the Stage 1 sub-corpus alone, and hold more strongly there.

### 3.5. Screening and Selection Process

Records from all databases were combined and de-duplicated before screening. Two reviewers independently screened all titles and abstracts against the eligibility criteria; disagreements were resolved by discussion and consensus, and any record not reconciled in the first round was arbitrated by the senior author using the full text. Full-text eligibility was not assessed in duplicate: the first author assessed all full-text reports against Table 3 and the second author checked every decision, with disagreements resolved by discussion. No inter-rater agreement statistic was calculated at either stage, because agreement was not recorded at the time; reproducibility rests instead on explicit criteria, consensus resolution, and the documented PRISMA screening flow and the study-level coding matrix (Supplementary Materials Table S1). Across the two database stages, 42 records were identified. After removing 6 duplicates, 36 records were screened on title and abstract, of which 6 were excluded; the 30 remaining reports were assessed in full, and 2 did not meet the full-text eligibility criteria, leaving 28 studies included from database searching. Citation searching identified two further eligible peer-reviewed studies, giving a final corpus of 30 studies. The complete flow is shown in the PRISMA 2020 diagram (Figure 1). The 6 title/abstract exclusions were screened out before full-text retrieval and were not itemized at the time, so they are reported as a count only; Section 10 records this and the single-reviewer full-text stage as limitations.

### 3.6. Evidence-Confidence and Reporting-Completeness Appraisal

Because the corpus spans conceptual, simulation, and empirical work, and because no validated risk-of-bias instrument transfers to engineering simulation studies (Section 3.1), a tailored appraisal was used. It has two components. The first is a ten-item reporting-completeness checklist, applied to every study, covering the reporting of the DT definition and its operationalization, the routing formulation, the method, baselines, validation setting, data transparency, metric completeness, scalability, and implementation considerations such as governance, privacy, and interoperability; the items are defined in Supplementary Materials Table S1. The checklist is a reporting-completeness aid, not a validated risk-of-bias tool, and was not converted into a numerical score used to weight the synthesis.

The second component assigns each study to an evidence-confidence category. To make that assignment reproducible, it is a function of three recorded inputs rather than a global judgement—the validation setting actually demonstrated, the clarity of DT operationalization, and the clarity of the routing formulation—and Table 4 states the resulting decision rule. Three further rules applied: maturity was assigned from the highest level of validation a study demonstrates, not the level it claims, so a study describing field deployment as future work remains simulation-only; a study meeting a category’s validation condition but not its reporting conditions took the lower category, so borderline cases resolve downward; and conceptual or secondary review sources cannot exceed “low”, because they report no validation of their own. The first author appraised every study and the second author re-checked each appraisal against the source article, with disagreements resolved by discussion. Appraisal results were used qualitatively and were not used to compute a weighted effect. No pooled quantitative effect estimate was attempted, because study designs, domains, performance metrics, and validation settings were heterogeneous.

### 3.7. Data Extraction and Coding

A structured template was used to extract, for each study: author and year; application domain; routing or problem type; DT role in the routing loop; coupling granularity; evidence maturity; evidence-confidence category; solver or learning method; data source; validation type; performance metrics; key contribution; and principal limitation. Four further fields are reported in Supplementary Materials Table S1: evidence type, DT level on the Kritzinger ladder, routing layer addressed, and whether the study reports any synchronization or data-freshness measure. Each was coded from the extracted record and re-checked against the source article.

The first author coded every study against written definitions of each category and the second author checked the full matrix against the source articles, with disagreements resolved by discussion. Coding is multi-label where the construct is multi-valued and single-label where it is not. DT role is multi-label, because monitoring, simulation, re-optimization, and infrastructure management routinely coexist in one system, so role counts sum to more than 30; coupling granularity is multi-label for the same reason. Application domain, evidence maturity, evidence type, and evidence confidence are single-label, and a study spanning two domains was assigned to the domain of the routing decision it evaluates. Borderline cases followed the same downward-resolving rules as the appraisal.

The evidence-type field most affects interpretation, since review articles are secondary evidence and cannot be read like primary studies. Of the 30 studies, 25 report primary evidence—19 simulation-based, 3 prototype or laboratory, 3 industrial or field—while 3 are conceptual frameworks and 2 are secondary reviews. The five non-primary studies are retained to map concepts, terminology, architectures, and gaps, and are used for nothing else: they never support a claim about algorithmic performance, validation maturity, or operational effectiveness, and wherever a count bears on operational validation, primary and non-primary studies are reported separately.

To move the synthesis beyond description, four analytical lenses were defined a priori: DT role (A, real-time monitoring and synchronization; B, simulation and training; C, adaptive decision-making and re-optimization; D, infrastructure, data governance, and model management); coupling granularity (object, vehicle, fleet/network, cyber layer); evidence maturity (conceptual, simulation-only, prototype/laboratory, industrial/field); and solver class (exact optimization, metaheuristics, reinforcement and deep reinforcement learning, multi-agent systems, model-predictive and control-based planning, hybrid AI–optimization). The study-level coding is in Supplementary Materials Table S1, with the cross-domain comparison summarized in Table 5.

### 3.8. Synthesis Method and Traceability

Synthesis proceeded in three steps. First, a thematic synthesis grouped studies into five application domains (Section 5). Second, a cross-case synthesis re-cut the same corpus along the four analytical lenses to identify mechanisms and trade-offs invisible within a single domain (Section 6). Third, recurring structural elements were consolidated into a routing-specific reference architecture and a set of evidence-informed design hypotheses (Section 7).

The second and third steps are where interpretation enters, so the procedure is stated explicitly. Studies coded in Supplementary Materials Table S1 were grouped by domain and routing task, then cross-tabulated pairwise across DT role, domain, coupling granularity, evidence maturity, and solver family, so that each lens could be read against the others. A pattern was recorded only where multiple studies supported it within a cross-tabulation cell, or where a property was repeatedly *absent* across domains—an absence being evidential for a reporting gap though not for a performance claim. Architecture components were derived from the routing-loop functions recurring across those patterns (sensing and state capture; synchronization and model update; simulation and prediction; optimization and re-optimization; decision execution; governance, security, interoperability, and model management), and hypotheses were formulated only where the corpus showed a recurring issue but supplied too little validation to settle it.

Because these steps mix observation with inference, every substantive statement in Section 4, Section 5, Section 6, Section 7 and Section 8 is meant to be read at one of four levels: (i) a study characteristic or validation setting reported directly by an included study; (ii) a cross-study pattern identified through the coding; (iii) a design requirement inferred from a repeated gap or system dependency; or (iv) a hypothesis for future testing. The traceability table in Section 7 makes the level explicit for the architecture components and hypotheses.

Throughout, evidence-confidence categories governed the strength of language used, so that field-validated evidence could support stronger statements while conceptual evidence informed only the research agenda. The synthesis is narrative and structured, and no meta-analytic pooling was performed. This was a methodological decision, not an omission: the studies differ in domain, routing formulation, solver family, benchmark instance, validation environment, and reported metric, so no common effect measure exists and few share a baseline. Pooling percentage improvements computed against different baselines, in different units, under different validation regimes would yield a precise number with no defensible interpretation; Section 10 records the resulting loss of quantitative comparability.

## 4. Descriptive Overview

The final corpus comprises 30 studies identified through the two-stage search and targeted citation searching described in Section 3. Publication years span 2021–2026, with a pronounced concentration in 2025–2026 (n=24); Figure 2 maps this temporal evolution, showing how the field broadened from early production- and AGV-centered work toward emergency, urban, supply-chain, cold-chain, electric-freight, platooning, IoV, and DT-infrastructure applications. The 2026 cohort introduces cold-chain multi-depot routing, traffic-aware electric-vehicle routing and charging, cooperative truck platooning, DT model migration, and DT wireless channels, which is best read as rapid expansion and diversification of applications rather than as methodological maturation. Maturation would require evidence of a shift toward more robust validation, and although all six prototype- and field-validated studies are 2026 publications, all six also entered through the Stage 2 update search (Section 3.4), so growth in the field and growth in the search cannot be separated within this corpus. The 2025–2026 concentration should therefore be read as reflecting both the rapid recent growth of the field and the broader terminology and domain coverage captured during the update search.

By domain, the corpus divides into production and intralogistics (n=7), emergency and healthcare logistics (n=2), urban mobility and smart transportation (n=7), supply chain, warehousing, cold chain, and energy-aware logistics (n=8), and IoV, V2V, communication routing, and DT infrastructure (n=6). Under the DT-role lens, adaptive decision-making and re-optimization (Role C, n=20) and real-time monitoring and synchronization (Role A, n=15) are the most frequently instantiated roles, followed by infrastructure and model management (Role D, n=10) and simulation/training (Role B, n=6); many studies instantiate more than one role, so role counts sum to more than 30 (an observation developed in Section 6). A concise cross-domain summary is given in Table 5, and the complete study-level coding in Supplementary Materials Table S1.

The evidence-maturity profile is the single most consequential descriptive finding. Of the 30 studies, 19 are simulation-only and 5 are conceptual architectures or secondary reviews, whereas only 3 reach prototype or laboratory validation and just 3 are validated against industrial or field data—namely, electric-truck energy modeling [22], vehicle-usage analytics [36], and cooperative truck platooning with real logistics data [37]. Read against the evidence-type field, 25 of the 30 studies contribute primary evidence and 5 do not, so claims about operational validation rest on 25 studies, of which 6 reach prototype level or above. This distribution indicates an evidence base that is broad in scope but dominated by simulation and conceptual studies, and it tempers any general claim that DT-enabled routing has shown field-level benefits at scale.

## 5. Thematic Classification of DT–VRP Research

The corpus is organized into five application domains that differ in data environment, uncertainty, and optimization objective. The classification is descriptive of where research clusters, and Table 5 summarizes the cross-domain comparison; the analytical comparison along the cross-cutting lenses follows in Section 6. Each subsection identifies the mechanism by which the twin contributes and, where relevant, distinguishes demonstrated results from conceptual proposals.

### 5.1. Production, Factory, and Intralogistics Routing

Production and intralogistics is the domain in which the routing decision is most explicitly formulated in this corpus, and the one with the tightest physical–virtual coupling. Pan et al. [6] formulate a DT-driven production-logistics synchronization system for a VRP with pick-up and delivery in an industrial park, using the twin to keep routing aligned with live shop-floor state. Zhang et al. [13] integrate AGV dispatching and routing through a DT coupled with deep reinforcement learning (DRL), training navigation policies within the twin before deployment, and Zhang et al. [40] address conflict-free path planning for multiple autonomous vehicles. The domain extends naturally to automated container terminals, where Lou et al. [15] couple a DT with an improved artificial-fish-swarm and Dijkstra solver to schedule and route AGVs while detecting and resolving conflicts through physical–virtual fusion. The newly integrated studies further sharpen how the twin participates in the decision loop: Li et al. [41] demonstrate object-level synchronization, using YOLOv8 vision and a Redis exchange to mirror packing boxes into a DT logistics system; Fu and Zhu [42] employ the twin as a reinforcement-learning environment for flexible assembly job-shop scheduling under dynamic material kitting; and Lee et al. [16] position the twin as a participant in both training and decision phases of dynamic AGV optimization, directly countering the common pattern in which a DT is used only for post-hoc validation. Two things should be kept apart here. The controlled industrial setting can supply high-frequency, high-fidelity sensor data, and that is a property of the application environment rather than a demonstrated property of the evidence: six of the seven studies in this domain are simulation-only and the seventh reaches prototype level, so sim-to-real transfer remains unproven and the domain’s apparent advantage is a design opportunity rather than a validated result.

### 5.2. Emergency and Healthcare Logistics

Emergency and healthcare logistics is the most uncertainty-driven and life-critical setting, where the objective shifts from efficiency to response time under disruption. Abdelnabi and Rabadi [9] formulate real-time medical-aid delivery as a dynamic VRP supported by a DT that ingests evolving incident and traffic information, and Ikram et al. [14] use a DT to assist real-time emergency-vehicle navigation, resource optimization, and reroute planning. In both cases, the twin combines synchronization (Role A) with adaptive re-optimization (Role C) to support rapid rerouting and triage. This domain also frames the clearest research need in the corpus: because decisions affect morbidity and mortality, opaque policies are harder to justify, yet neither study reports any trust or explainability measure and both remain simulation-based. Explainability is therefore identified here as an evidence-based gap—a property that the setting demands and that no study in the corpus supplies—and not as an empirical finding of the reviewed work. The scarcity of studies relative to the stakes involved is itself a notable gap.

### 5.3. Urban Mobility and Smart Transportation

Urban mobility is the most heterogeneous domain and the one that grew most within this corpus: six of its seven studies are 2026 publications retrieved in the Stage 2 update, so its prominence reflects recent activity and the update search together rather than growth alone. It is characterized by large-scale, noisy, multi-source sensor data. Chen et al. [8] couple a DT with spatiotemporal learning for sequential travel-time prediction and intelligent routing. The new studies broaden the domain along several axes. On energy-aware mobility, Xie et al. [18] present a DT-based approach for dynamic, traffic-aware electric-vehicle routing and charging that continuously synchronizes with the network and uses a dual-population evolutionary algorithm, reporting reductions in travel time and energy use while remaining robust to traffic disruptions, road closures, and charging-station failures. On traffic control, Lis and Mądziel [17] combine a calibrated microsimulation (validated with the GEH statistic) with adaptive inflow-metering control, reaching prototype maturity through software-in-the-loop testing; Wang et al. [43] contribute a roadside LiDAR–camera fusion benchmark for urban traffic-flow twins; and Yang et al. [44] develop DT-based trajectory planning with improved A* and model-predictive control. On cooperative and analytic mobility, Guo et al. [37] propose a DT-enabled cooperative autonomous truck-platooning framework verified with real logistics data, and Rubio et al. [36] describe a vehicle-usage DT pipeline validated on field data—two of the few field-grounded studies in the corpus. Related background literature points in similar directions, including a non-peer-reviewed proof-of-concept DT for cooperative autonomous car-sharing [38] and a conceptual account of DTs for resilient cities [39]. These sources are discussed for context only and are not used to support any conclusion about the performance or validation maturity of the 30 included studies. The domain’s defining challenge is scaling high-fidelity twins to city scale while controlling sensor noise and computational cost.

### 5.4. Supply Chain, Warehousing, Cold Chain, and Energy-Aware Logistics

Supply-chain-level work extends the twin beyond the route to multi-echelon coordination, and it is here that the newly added studies most directly address earlier coverage gaps. It is also the domain in which primary and secondary evidence are most easily confused, so the distinction is made explicitly. Earlier studies are largely system-level or conceptual: Marmolejo-Saucedo [19] applies a DT to large-scale supply-chain optimization through a packing problem; Vashishth et al. [2] survey DT solutions for smart logistics; and Abideen et al. [1] position DT-integrated reinforcement learning for logistics decision support as a conceptual framework. The 2026 additions introduce concrete routing-relevant capability. Alam et al. [20] review AI-enhanced DTs for warehouse logistics and explicitly flag return-on-investment, sustainability, and reverse-logistics gaps. Nozari and Yordanova [21] present an adaptive fuzzy multi-objective DT for multi-depot cold-chain VRP, in which the twin updates uncertain parameters and re-optimizes routes under temperature and time-window constraints. Kaarlela et al. [22] couple gradient-boosting energy modeling, trained on 180 real electric-truck trips, with route choice and charging-infrastructure scenario evaluation, providing rare field-grounded evidence for energy-aware freight routing. Jiang et al. [45] address a routing-critical logistics node, using a DT and improved RRT for port inspection-robot path planning, and Chen et al. [46] propose an AI-driven DT architecture for market-volatility warning that foregrounds missing DT standards and AI governance. Of the eight studies in this domain, four report primary evidence, two are secondary reviews [2,20], and two are conceptual frameworks [1,46]; the reviews and the frameworks are used here to map gaps and terminology and are not counted toward any claim about routing performance. On that basis, the domain has moved from conceptual breadth toward specific, sustainability- and governance-aware routing problems, though several of the primary studies remain case-bounded.

### 5.5. IoV, V2V, Communication Routing, and DT Infrastructure

The IoV domain extends the review to cyber-layer routing mechanisms that support, constrain, or synchronize vehicle-routing decisions. These are not equivalent to physical vehicle routing: their decision variables, constraints, and objectives concern the movement of information and computation rather than of goods, and they are treated throughout as enabling infrastructure for the routing loop rather than as instances of it. Alam et al. [23] present multi-agent best-routing in high-mobility DT-driven IoV; Cong Dao et al. [7] develop metaheuristic multi-hop routing for IoT-based DTs; Zhang et al. [47] analyse V2V time-varying complex networks; and Zahangir Alam et al. [48] propose graph-assisted DT-driven multi-agent shared offloading. The new studies add explicit DT-infrastructure capabilities: Zhang et al. [34] set out the architecture, technologies, and standardization directions for a digital twin of wireless channels supporting ITS communication reliability, and Xing et al. [24] introduce fine-grained, model-level DT migration (FGDT), treating the twin as a decomposable sub-model ensemble and optimizing its migration with a proximal-policy-optimization strategy to reduce synchronization and communication overhead. These studies predominantly instantiate Role D (infrastructure and model management) at the cyber layer. Their significance for physical routing is that they identify the reliability and placement of the twin as candidate determinants of routing performance in distributed deployments. The corpus suggests that these may be important determinants, but it does not establish it: no study in the corpus varies twin reliability or placement while holding the routing problem and solver fixed, so the comparative evidence needed to rank them against other determinants does not yet exist.

## 6. Cross-Theme Analytical Synthesis

Grouping by domain shows where research clusters but not how digital twins create value. This section re-cuts the 30 studies along four analytical lenses—DT role, coupling granularity, evidence maturity, and solver class—and then examines evaluation practice. The central argument is that the taxonomy suggests part of the apparent fragmentation of DT–VRP research arises from conflating fundamentally different DT roles, and that separating them makes coherent design insight visible. Role conflation is not claimed to be the only or the principal cause: differences in application domain, routing formulation, optimization objective, data environment, system scale, and validation maturity plainly contribute as well, and the analysis presented here does not apportion between them.

### 6.1. DT Role Taxonomy

Across the corpus, the twin performs four distinguishable roles in the routing loop (Figure 3). In Role A—real-time monitoring and synchronization (n=15), the twin mirrors routing-relevant state and the optimizer reacts to a synchronized picture, exemplified by vision-synchronized intralogistics objects [41] and emergency-vehicle state tracking [14]. In Role B—simulation and training (n=6), the twin is a safe environment for learning and scenario testing [13,16]. In Role C—adaptive decision-making and re-optimization (n=20), the twin updates uncertain parameters and re-solves the routing problem in a closed loop, as in cold-chain multi-objective routing [21] and traffic-aware EV routing [18]. In Role D—infrastructure, data governance, and model management (n=10), the twin is operational infrastructure data pipelines, cyber-risk monitoring, wireless-channel twins, and model migration [24,34,46]. Because many studies combine roles, the counts sum to more than 30 and the taxonomy is a lens on functions rather than a partition of papers; its value is in clarifying that a claimed DT benefit means different things in different roles, which is why cross-study comparison has historically been difficult.

Two points of scope should be stated. First, Role D groups functions that manage the information infrastructure a routing-oriented twin depends on: data governance, communication reliability, cyber-risk monitoring, and model migration. They are grouped because each affects the availability, trustworthiness, or deployability of the routing decision loop, not because they operate at the same technical layer—they plainly do not, and a larger corpus would justify splitting Role D into a data-governance and a distribution/migration sub-role. Throughout the analysis, Role D is therefore treated as a broad enabling-infrastructure category. Claims derived from data-governance studies are not generalized to communication, security, or migration functions unless those subfunctions are directly supported by the cited studies. Second, the four lenses were defined a priori (Section 3.7) as an analytical classification imposed on the corpus in order to examine it, and are not presented as a taxonomy that emerged inductively from the evidence. What the corpus contributes is the distribution of studies across the lenses and the cross-tabulations between them; the lenses themselves are the authors’ instrument.

### 6.2. Coupling Granularity

The lens of coupling granularity—what, precisely, is twinned—cuts across domains and provides a perspective for examining potential differences in fidelity, sensing cost, computational burden, and scalability. Object-level coupling (n=4) twins individual items such as packing boxes or perception targets and supports the finest synchronization [41,43]. Vehicle-level coupling (n=13) twins individual vehicles or AGVs for navigation and trajectory control [15,44]. Fleet/network-level coupling (n=17) twins interacting fleets or whole networks for system-level routing and repositioning [8,18,22]. Cyber-layer coupling (n=7) twins the communication and computation substrate itself [7,24,34].

Two qualifications matter for how this lens is read. First, the four categories are not four points on one granularity scale. Object, vehicle, and fleet/network coupling do form a nested progression over physical entities, but cyber-layer coupling is a distinct system dimension: it twins the communication and computation substrate rather than a coarser aggregation of vehicles, and a system can also couple at the cyber layer and at a physical level simultaneously; the coupling categories are therefore non-exclusive. Second, the widely repeated expectation that finer granularity buys fidelity at the cost of sensing and computation, while network- and cyber-layer twins trade per-entity detail for scalability, is consistent with the corpus but is not demonstrated by it: no included study measures fidelity, sensing cost, computational load, and scalability on a common basis, and none compares granularities within a fixed routing problem. The trade-off is therefore stated here as a design expectation that the corpus is compatible with and that Section 8 treats as untested, rather than as a cross-study finding.

### 6.3. Evidence Maturity and Validation Strength

Mapping the corpus against validation strength (Figure 4) quantifies the maturity pattern introduced in Section 4. Simulation-only studies dominate (n=19), conceptual and secondary studies are common (n=5), prototype or laboratory validation is less frequent (n=3), and industrial or field-data validation is rare (n=3). Maturity is unevenly distributed across domains: production and IoV work is almost entirely simulation-based, emergency logistics is exclusively so, and the few field-grounded studies cluster in urban mobility and energy-aware freight [22,36,37]. Much of the reported performance benefit of DT-enabled routing is therefore demonstrated in silico, and claims of operational advantage should be stated conditionally until corroborated by field evidence. This also identifies the clearest near-term contribution available to the field: moving representative simulation studies up the maturity ladder through prototype and field validation.

### 6.4. Algorithms, Solvers, and Learning Methods

The corpus draws on a wide methodological palette (Table 6). Hybrid AI–optimization methods are the most common family (n=9), combining learning, fuzzy modeling, or evolutionary search with optimization or control [18,21,22,37]. Reinforcement and deep reinforcement learning form the most prominent single learning approach (n=5), typically paired with a DT acting as the training environment [13,16,42]. Multi-agent systems (n=2) and model-predictive or control-based planning (n=2) appear mainly in IoV and vehicle-level control, respectively, while metaheuristics and heuristics (n=4), learning-based perception and analytics (n=3), and exact optimization (n=1) each appear in a smaller number of studies; the remaining conceptual, review, and network-analysis contributions (n=6) propose architectures without committing to a single solver.

Table 6 should be read as a map of methodological practice, not as a comparison of solver effectiveness. The families are evaluated on different problems, in different domains, against different baselines, in different validation environments, and with different reported metrics, and no study in the corpus compares families on a common instance. Any apparent ranking between families is therefore an artefact of where each family happens to have been applied, and the corpus supports no claim that one family outperforms another. The same heterogeneity is what precludes pooled synthesis (Section 3.8).

### 6.5. Performance Metrics and Evaluation Gaps

Evaluation practice is heterogeneous and only partially comparable across studies. Distance, travel time, and cost recur in physical-routing work; response time dominates emergency logistics; latency, overhead, and delivery ratio dominate IoV; and energy, emissions, and service-level metrics appear mainly in the newer sustainability-oriented and cold-chain studies [18,21,22]. Three evaluation gaps are evident. First, synchronization quality and data freshness—arguably the defining DT property, and the property most directly determined by the underlying sensor and communication infrastructure—are rarely measured: only 2 of the 30 studies report an explicit synchronization measure and a further 8 report a proxy such as communication or migration latency, leaving 20 with none (Supplementary Materials Table S1), so their contribution cannot be isolated from that of the solver. Second, robustness to disruption and to twin–reality divergence is seldom quantified, despite being central to the value proposition. Third, explainability and trust are essentially unmeasured, even in safety-critical settings. The absence of shared benchmarks and reporting conventions for these properties is a primary obstacle to cumulative progress and is taken up in the research agenda (Section 9).

Because this gap is the one most often raised against DT-enabled routing research, Table 7 sets out a concrete set of measurable indicators that would close it. These are proposed evaluation variables, not properties measured by the reviewed studies; the corpus reports almost none of them, which is precisely the finding. The set is deliberately drawn from quantities that an instrumented DT-routing system already has access to, so that reporting them requires additional discipline rather than additional sensing. Several have established analogues elsewhere—state staleness is the Age of Information of the status-update literature [49], and update latency and communication reliability are standard networking measures—which makes them tractable to standardize.

## 7. Reference Architecture and Design Hypotheses

The cross-case synthesis recurs across domains as a small set of structural elements that can be organized as a control loop. Figure 5 presents these as a routing-specific reference architecture. The architecture is not proposed as a new generic digital-twin architecture; layered DT stacks are well established [11,12]. Its contribution is to translate generic DT layers into a routing-specific control-loop model—organized around routing-state variables, routing decisions, and routing-specific bottlenecks—and to mark where the reviewed studies currently provide strong, moderate, or weak evidence.

Because a reference architecture is the output most exposed to the objection that it reflects the authors’ judgement rather than the corpus, Table 8 states, for each component and hypothesis, the coding dimension that supports it, the domains and studies in which it appears, the maturity of that evidence, and its epistemic status on the four-level scale of Section 3.8: directly demonstrated in multiple studies, a cross-study pattern, a design requirement inferred from a repeated gap, or a hypothesis for future testing. Three components—synchronization quality as a measured property, decision latency as an explicit constraint, and human oversight—are inferred from repeated absence rather than observed presence, and two—DT distribution and migration, and communication-state representation—rest predominantly on cyber-layer studies and are scoped accordingly.

The physical layer comprises the routing-relevant entities and their environment: vehicles, AGVs, trucks, ambulances, and autonomous cars; depots, customers, roads, charging stations, ports, and warehouses; and the sensors that generate telemetry. Across the corpus, this layer ranges from individual items [41] to container terminals [15], city traffic [17], and electric-truck fleets [22]. Step (1) of Figure 5 is the constraint this layer imposes on the next: sensing coverage and data quality bound everything downstream, since no synchronization procedure can recover state that was never observed.

The data and synchronization layer carries the bidirectional flow between physical operations and the twin: GPS and IoT telemetry, traffic feeds, energy state, orders, and V2X messages, with sensor fusion and high-frequency state synchronization. The corpus motivates treating synchronization quality and data freshness as explicit, measurable properties of this layer (Table 7), and treating model management—including DT distribution and migration—as a deployment-conditioned architectural component, particularly for distributed, edge–cloud, IoV, and geographically distributed implementations [24,34]. The scope of that last element should be stated precisely. Twin distribution and migration are derived almost entirely from cyber-layer studies, and they become central concerns for distributed and edge–cloud implementations—IoV fleets, city-scale deployments, and any system in which the twin does not reside where the decision is taken. For a single-site deployment with a co-located twin, such as an intralogistics DT confined to one shop floor, they are not first-order, and the architecture should be read as scoping them to deployment conditions rather than asserting them universally.

The twin maintains the routing-state variables on which decisions depend: vehicle state, demand state, network state, energy and charging state, communication state, and an explicit uncertainty state, together with a scenario simulator for evaluating candidate actions before they are taken. Representing uncertainty and communication state as first-class routing variables—rather than as external context—is a design proposal by the authors, not a structure that emerged consistently from the reviewed studies. Its basis is asymmetric evidence: the reviewed studies populate vehicle, demand, network, and energy state to varying depths [8,18,21], whereas uncertainty is represented explicitly in only a small number of studies—most clearly in the fuzzy multi-objective cold-chain formulation of Nozari and Yordanova [21]—and communication state is treated as a decision variable almost exclusively in the cyber-layer studies [7,23,34]. The proposal is therefore that these two variables be made explicit because their absence recurs, not because the corpus demonstrates the benefit of making them explicit.

The decision layer converts twin-derived state into routing-specific outputs: generated routes, dispatch decisions, re-routing, charging schedules, fleet and platoon coordination, emergency rerouting, and multi-hop cyber routing. It draws on exact and dynamic VRP solvers, metaheuristics, reinforcement learning, multi-agent coordination, and model-predictive control. The architecture makes decision latency and solver feasibility explicit constraints between the twin and the decision layer, because a route that is optimal but late is operationally useless. This too is an inferred requirement rather than an observed practice: real-time responsiveness is invoked as a motivation across the corpus but is reported as a measured quantity in only a handful of studies, so making it an explicit cross-layer constraint responds to a reporting gap rather than codifying established practice.

The layers are connected through a closed-loop decision process comprising sensing, state synchronization, simulation, optimization, route dispatch or re-routing, and subsequent monitoring. Field validation and monitoring provide feedback to the physical operations layer, as represented by step (4) in Figure 5. This closed-loop formulation makes several routing-specific limitations more explicit, including synchronization latency and state staleness, solver runtime under real-time constraints, model drift between the digital twin and the physical system, migration overhead in distributed deployments, uncertainty representation, and the role of human intervention. These operational and methodological constraints, rather than the layered structure alone, constitute the principal analytical value of the proposed architecture.

Five considerations cut across the layers and are represented as a cross-cutting panel in Figure 5: interoperability and open standards; cybersecurity and data privacy; explainability; human oversight; and DT migration and model management, with field validation as the closing feedback. The corpus shows these to be widely acknowledged but rarely operationalized [34,46], and that is the limit of what it shows. They are therefore stated here as design considerations whose importance depends on deployment conditions, not as universal requirements that every DT-routing system must satisfy: explainability becomes material where decisions are safety-critical or regulated; privacy and security where the data are personal or the network is exposed; interoperability where a twin must exchange state across organizational boundaries; human oversight where accountability for a decision rests with a person; and DT migration and model management where the twin is distributed across edge and cloud. No study in the corpus evaluates a system with and without any of these capabilities, so their contribution is unquantified, and Table 8 records each as an inferred requirement rather than a demonstrated one.

The synthesis generates three design hypotheses. They are stated as hypotheses for future testing, not as established results, because the corpus is dominated by simulation-only evidence and rarely reports the metrics that would confirm them. The interpretive-status label attached to each hypothesis describes the strength of the pattern behind it; it is not a study-level evidence-confidence rating in the sense of Section 3.6.

**Hypothesis** **1.** *Synchronization quality and data timeliness are expected to influence routing performance in DT-enabled systems; their relative importance compared with solver choice remains an empirical question requiring controlled comparative evaluation. Evidence basis: studies in which the twin participates in the decision loop with explicit state alignment [16,24,41], against a broadly similar solver palette across systems (Section 6.4). Interpretive status: hypothesis with limited provisional support; the mechanism is plausible but directly untested, and because synchronization quality is measured in only 2 of the 30 studies, the corpus cannot separate its contribution from that of the solver. To test: controlled experiments that vary synchronization latency and update frequency while holding the solver fixed, and the converse experiment holding synchronization fixed while varying the solver. Required metrics: the indicators in Table 7, in particular, state staleness, update frequency, and twin–reality divergence, reported alongside routing cost and time.*

**Hypothesis** **2.** *Model management, DT migration, interoperability, and governance are expected to improve the scalability, deployment robustness, and maintainability of DT-enabled routing systems, particularly in distributed and edge–cloud implementations. Whether they are necessary conditions for scaling beyond proof-of-concept is not established by this corpus. Evidence basis: the maturity profile (Figure 4) and the observation that deployment-oriented studies add model-management or migration capability [24] or field validation [22,37]. Interpretive status: hypothesis with provisional support. This is not a study-level evidence-confidence rating as defined in Section 3.6. The association is visible in deployment-oriented studies, but no included study compares otherwise similar systems with and without these capabilities. To test: scaled deployments comparing systems with and without model-management and interoperability layers. Required metrics: migration and communication overhead, scalability (fleet or node count), standard compliance, and deployment cost.*

**Hypothesis** **3.** *Jointly modeling energy, infrastructure, and operational constraints within the routing loop is expected to improve the reliability of sustainability-oriented routing decisions relative to evaluating emissions only as a post hoc indicator, measured through the consistency between predicted and observed energy use, charging feasibility, service-level compliance, and emissions or cost outcomes. Evidence basis: studies that co-model energy and charging within routing report concrete routing consequences [18,21,22], including field data for energy modeling. Interpretive status: hypothesis with provisional support. To test: comparison of co-modeled and post hoc emissions formulations on the same network. Required metrics: predicted-versus-observed energy consumption, emissions, charging feasibility and utilization, service level, and cost.*

## 8. Discussion

The synthesis surfaces a set of recurring tensions that define the current discourse. Each is a design trade-off rather than a settled question, and each maps onto the architecture of Section 7.

They should be read at the four levels set out in Section 3.8, and most of what follows sits at levels (ii) and (iii)—cross-study patterns and inferred design requirements—rather than at level (i). Because the corpus is dominated by simulation studies, holds only three field-validated studies, and rarely measures the DT properties at issue, the discussion is deliberately conditional: it identifies where each trade-off arises and what would settle it, rather than resolving it.

A central tension concerns whether routing should be optimized centrally, by a controller that aggregates all state, or in a decentralized fashion, by agents acting on local information. Centralized twins can support system-wide optimization but face scalability bottlenecks and sensitivity to communication delay; decentralized twins offer scalability and resilience, especially in IoV [23,48]. Neither property is intrinsic to the architecture. Centralization does not by itself guarantee system-level optimality, nor does decentralization by itself entail its loss: both depend on the optimization formulation, the information structure available to each decision-maker, the coordination mechanism, and the solution method, and a decentralized scheme with adequate coordination can match a centralized one on some formulations. The model-migration work of Xing et al. [24] occupies a productive middle ground, distributing twin sub-models to balance overhead against coordination. The evidence does not favor one pole; the appropriate choice depends on the formulation, the coupling granularity, and the synchronization quality achievable in a given deployment.

A second tension concerns model fidelity. High-fidelity, physics-based twins can support more accurate prediction but impose computational cost that may compromise real-time responsiveness, whereas lightweight twins are faster but less faithful [8,9,43]. Fidelity alone does not guarantee accuracy: a high-fidelity model that is poorly calibrated, or validated only against the simulator that generated its inputs, can be less accurate in deployment than a simple model fitted to field data, so calibration and validation matter at least as much as model resolution. The corpus suggests this is best treated as adaptive rather than fixed: the required fidelity follows from the DT role and coupling granularity, so that object-level monitoring and city-scale repositioning warrant different fidelities. Adaptive-fidelity twins that vary resolution by context remain an open and largely unaddressed design problem.

The growing reliance on reinforcement and deep learning raises the tension between performance and transparency. High-performing learned policies are often opaque, which is problematic in safety-critical emergency and autonomous-mobility settings [14,44]. The review found essentially no quantitative reporting of explainability or trust, even where the application demands it. Explainable, auditable routing policies are therefore identified here as a methodological gap in the corpus and as a design requirement that is likely to become important where routing decisions are safety-critical or subject to regulatory oversight. The corpus does not establish that explainability is a precondition for deployment, and no included study evaluates a deployment in which it was required; the claim is a judgement about regulatory trajectory, not a finding of this review. Section 9.1 sets out what an explainability roadmap for DT-enabled routing would need to contain.

Most systems are highly domain-specific, which yields strong in-domain performance but limits transfer. The role taxonomy and reference architecture are offered as a response: expressing diverse systems in a common vocabulary of roles, coupling, and layers makes cross-domain comparison and reuse tractable without forcing premature standardization of solvers. The persistence of domain silos, in which few studies cite across domains, suggests the binding constraint is conceptual and organizational as much as technical.

Because DT–VRP systems depend on continuous, often sensitive sensor data—traffic flows, patient locations, vehicle telemetry—questions of ownership, privacy, cybersecurity, and interoperability arise as design considerations, particularly in IoV and healthcare [7,34]. Yet the corpus treats these issues largely conceptually; Chen et al. [46] explicitly note the absence of DT-specific standards and AI-governance mechanisms. A compromised or spoofed telemetry stream corrupts the twin state and, through it, every downstream routing decision, so sensor-data integrity is a routing-safety property, not only an IT concern. Interoperability is best understood in the same conditional terms: shared data models and standards are likely to be important wherever twins must exchange state across organizational boundaries, and the corpus supplies no evidence that they are strictly necessary in single-organization deployments. The stronger claim—that scalable cross-organizational ecosystems cannot materialize without them—is a design judgement, and the corpus neither tests nor supports it.

The newer studies push three societally important concerns to the foreground. Sustainability appears to become actionable in practice when energy, emissions, and infrastructure are modeled jointly with routing rather than evaluated afterwards [18,21,22]; this is the proposition stated as Hypothesis 3 and it requires empirical validation, so it is not presented here as a settled conclusion. Resilience requires twins that anticipate disruption beyond routine congestion [39], a direction indicated by background literature outside the corpus rather than by an included study. Human oversight—keeping a human meaningfully in or on the loop—remains aspirational across the corpus despite its importance for trust and accountability. These concerns constitute the cross-cutting considerations of the reference architecture, and their underdevelopment is the clearest signal of where the field must mature.

## 9. Future Research Agenda

The agenda follows directly from the gaps identified in the synthesis and from the three design hypotheses, organized by time horizon in Figure 6 and detailed, with their evidential basis, in Table 9. It is deliberately evidence-linked: each direction names the finding that motivates it, so that priorities can be revised as the evidence base matures.

In the short term, the field would benefit most from measurement discipline: standardized DT–VRP benchmarks and explicit reporting of synchronization quality and data freshness along the lines of Table 7 (Hypothesis 1), explainable-AI methods for safety-critical routing, and richer sensor fusion that extends DT coverage to inventory and warehousing. In the medium term, the priorities are model management and migration to move systems beyond proof-of-concept (Hypothesis 2), sustainability-aware routing that couples energy, emissions, and infrastructure (Hypothesis 3), and end-to-end supply-chain integration with disruption-aware, resilient routing. In the long term, the field should pursue interoperable, cross-organizational DT ecosystems with open standards, the convergence of physical and cyber routing in unified twins, and human-centered governance and trustworthy autonomy at deployment scale.

### 9.1. An Explainability Roadmap for DT-Enabled Routing

Explainability is invoked throughout this literature and operationalized nowhere in it, so a concrete roadmap is more useful than a further statement of the gap. Nothing in what follows is implemented by any included study; it is a specification of what would need to be built and reported. For reinforcement-learning-based routing, the tractable targets are policy explanation that attributes a chosen action to the state features that drove it, reward decomposition that separates the contribution of travel time, energy, service level, and constraint penalties to a decision, counterfactual route alternatives that state what would have been chosen under a different state, and explicit constraint-violation explanation when a policy declines a feasible-looking route. For optimization and metaheuristic solvers, explainability is largely a reporting discipline: transparent statement of the objective and constraint set actually solved, sensitivity analysis over the parameters the twin supplies, and a decision log that records the inputs and the incumbent solution at each re-optimization. For safety-critical routing, the additional requirements are calibrated uncertainty rather than point predictions, a defined human-in-the-loop override with a recorded rationale, scenario-based validation over the disruption classes the system claims to handle, and a post-decision audit trail sufficient to reconstruct why a route was issued. For DT systems specifically, an explanation should be traceable along the loop of Figure 5: from the sensed state, through the synchronized twin state and the predicted scenario, to the selected route and its expected performance and risk. An explanation that stops at the solver omits precisely the part of the system a digital twin contributes.

### 9.2. Edge–Cloud Synchronization and the Security of Routing-Relevant DT Data

The synchronization gap identified in Section 6.5 has a systems dimension that the corpus touches only through its cyber-layer studies. Where a twin is distributed across edge and cloud, the split shapes update latency, computation cost, privacy exposure, resilience to partial failure, and the scale at which the system remains tractable, and can therefore condition the routing decision loop directly rather than incidentally. Three directions outside the corpus are relevant here and are noted as context rather than as reviewed evidence. Adaptive model splitting, in which the twin is decomposed and its parts placed to balance update latency against cost, is addressed both by the within-corpus migration work of Xing et al. [24] and by work on dynamic twin updating under unreliable and distorted inputs [50]. Federated and collaborative twin construction from distributed sensing, in which multiple edge nodes build partial twins that are integrated centrally [51], offers a route to city-scale twins without centralizing raw sensor data. These questions are most consequential for IoV, platooning, emergency routing, and city-scale deployments, and least so for single-site intralogistics twins.

Security belongs in the same discussion for a specific reason. The integrity and availability of the wireless links that carry twin updates bound the trustworthiness of every routing decision downstream of them, so an adversary who degrades or falsifies those updates degrades routing without ever touching the optimizer. Adversarial interaction in V2X and IoV settings is amenable to game-theoretic treatment, including at the physical layer, where the strategic interaction between legitimate transmitters, jammers, and eavesdroppers can be modeled explicitly [52]. Such work is not DT- or routing-specific and none of it met the inclusion criteria of this review; it is cited here to indicate a methodological direction for securing the synchronization path, not as evidence about routing performance. The corresponding gap within the corpus is more basic: no included study evaluates routing performance under adversarial or degraded communication conditions.

## 10. Limitations

The following boundaries define how far the findings can be interpreted. First, the review protocol was not prospectively registered. This is mitigated by full reporting of the search strategy, screening logic, eligibility criteria, PRISMA flow, appraisal procedure, data-extraction template, and supplementary coding matrix, and future updates should deposit the protocol in an open repository before screening. It also means that the Stage 2 search (Section 3.4) is reported as a documented iterative refinement rather than as a pre-specified amendment, and a reader should weigh it as such. Second, the search was restricted to English-language records in four sources, complemented by citation searching; relevant non-English or regionally indexed studies may not be represented, and the four sources differ in coverage and reproducibility in the ways set out in Table 2.

Third, three reporting gaps in the search and screening record should be stated plainly. The calendar date on which each source was last searched was not recorded and has not been reconstructed, so PRISMA item 6 is only partly satisfied. The reasons for the six records excluded at title and abstract were not itemized at the time of screening and are reported as a count only, so PRISMA item 16b is only partly satisfied for that stage. And although the two records identified through citation searching are included in the corpus, they were not flagged individually in the extraction record, so the Stage assignment in Supplementary Materials Table S1 reflects publication window rather than the retrieval route of those two studies. None of these values has been estimated or reconstructed. Fourth, full-text eligibility was assessed by one reviewer and checked by a second rather than assessed independently in duplicate, and no inter-rater agreement statistic was computed at either screening stage because agreement was not recorded; reproducibility rests instead on explicit criteria, consensus resolution, the PRISMA screening flow, and the study-level coding matrix.

Fifth, because the topic is fast-moving, several 2026 studies were early-access or only recently indexed at the time of searching. Sixth, the corpus is heterogeneous in design, domain, performance metrics, and validation setting, which precludes meta-analysis or pooled effect estimation; the synthesis is therefore narrative and structured, and the review consequently cannot state how much a digital twin improves routing performance on average, only under what conditions and with what evidential support improvements have been reported. Seventh, most of the evidence is simulation-based, so the review characterizes the state of the evidence and the plausibility of mechanisms rather than field-level effectiveness, and the design hypotheses are framed accordingly. Eighth, 5 of the 30 included studies are conceptual frameworks or secondary reviews rather than primary studies. They are retained for concept and gap mapping and are excluded from any claim about operational performance, but their presence means that counts over the full corpus should be read alongside the evidence-type field in Supplementary Materials Table S1. Ninth, some cyber-routing studies are conceptually adjacent to, rather than directly equivalent to, physical vehicle routing; they were included because cyber-layer routing is increasingly necessary for DT-enabled mobility, and their role is delimited explicitly in the eligibility criteria (Section 3.3), the role taxonomy, and the reference architecture.

## 11. Conclusions

This systematic review consolidated 30 peer-reviewed studies on digital twin-enabled vehicle routing across production and intralogistics, emergency and healthcare logistics, urban mobility, supply-chain and energy-aware logistics, and IoV and DT infrastructure, and is reported in accordance with PRISMA 2020. By coding each study for the role of the twin, its coupling granularity, the type and maturity of its evidence, and the routing layer it addresses, the review indicates that part of the field’s apparent fragmentation is attributable to four distinct DT roles—monitoring and synchronization, simulation and training, adaptive re-optimization, and infrastructure and model management—being compared as though they were the same thing, alongside differences in domain, formulation, data environment, and validation maturity.

Three conclusions follow. First, the evidence base is broad but empirically immature: simulation-only and conceptual studies dominate, 25 of the 30 studies contribute primary evidence and only 6 of those reach prototype level or above, and field validation is rare, so claims of operational benefit should remain conditional. Second, the determinants of DT–VRP performance may be as much architectural as algorithmic: synchronization quality, sensor-data freshness, model management, and the joint modeling of sustainability constraints are plausible candidates alongside the choice of solver, but because synchronization quality is measured in only 2 of the 30 studies the corpus cannot rank them, and they are therefore carried forward as the three evidence-informed hypotheses of Section 7 rather than as findings. Third, the cross-cutting concerns of governance, interoperability, explainability, resilience, and human oversight are consistently underdeveloped, and while the corpus cannot show that any of them is a necessary condition for deployment at scale, their absence is the most consistent gap it contains.

The review’s contribution is therefore synthetic rather than technical: a DT role taxonomy, an evidence-maturity mapping, and a routing-specific reference architecture with evidence-informed hypotheses that together provide a common vocabulary for comparing, designing, and evaluating DT-enabled routing systems, with the derivation of each architectural component and hypothesis traced back to the study-level coding in Table 8. By linking a concrete research agenda to the underlying evidence, it aims to support a transition from fragmented proofs-of-concept toward adaptive, sustainable, and human-centered routing systems validated in the field rather than only in simulation; realizing that transition will require coordinated progress on benchmarking, deployment, and governance as much as on optimization.

## Figures and Tables

**Figure 1:**
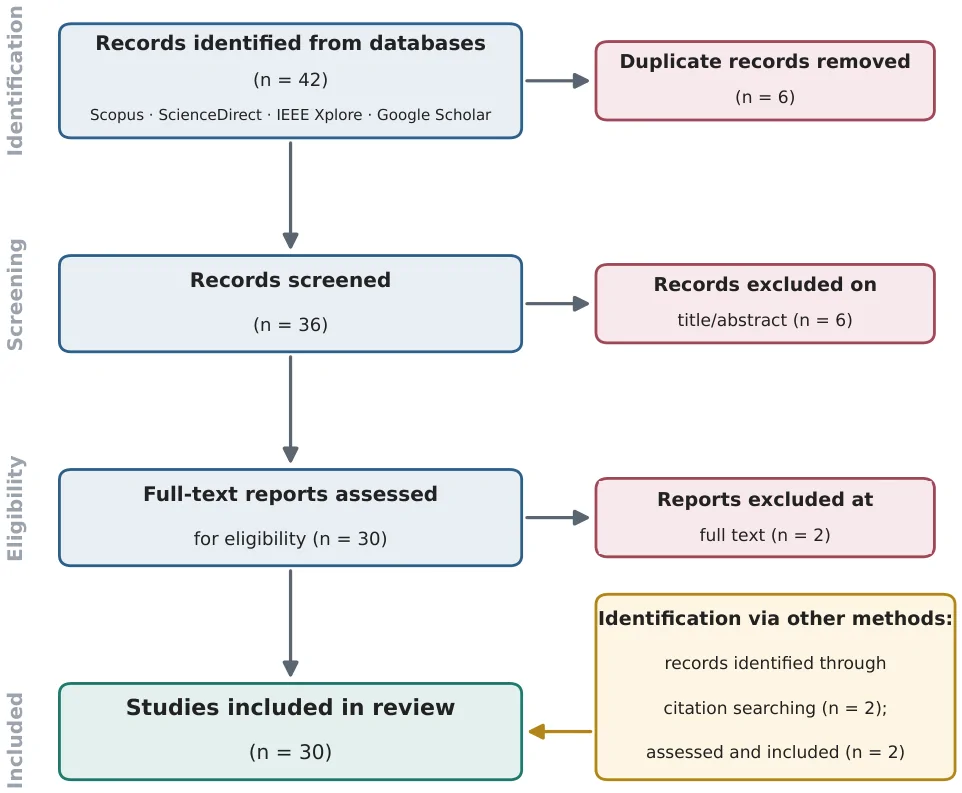
PRISMA 2020 flow diagram showing how the corpus was assembled. Database searching identified 42 records; after 6 duplicates were removed, 36 records were screened and 6 were excluded on title and abstract; 30 full-text reports were assessed and 2 were excluded under the eligibility criteria, leaving 28 studies; 2 further peer-reviewed studies were added through citation searching, giving a final corpus of 30 studies. The diagram documents the selection process and is a reporting device, not an analytical result.

**Figure 2:**
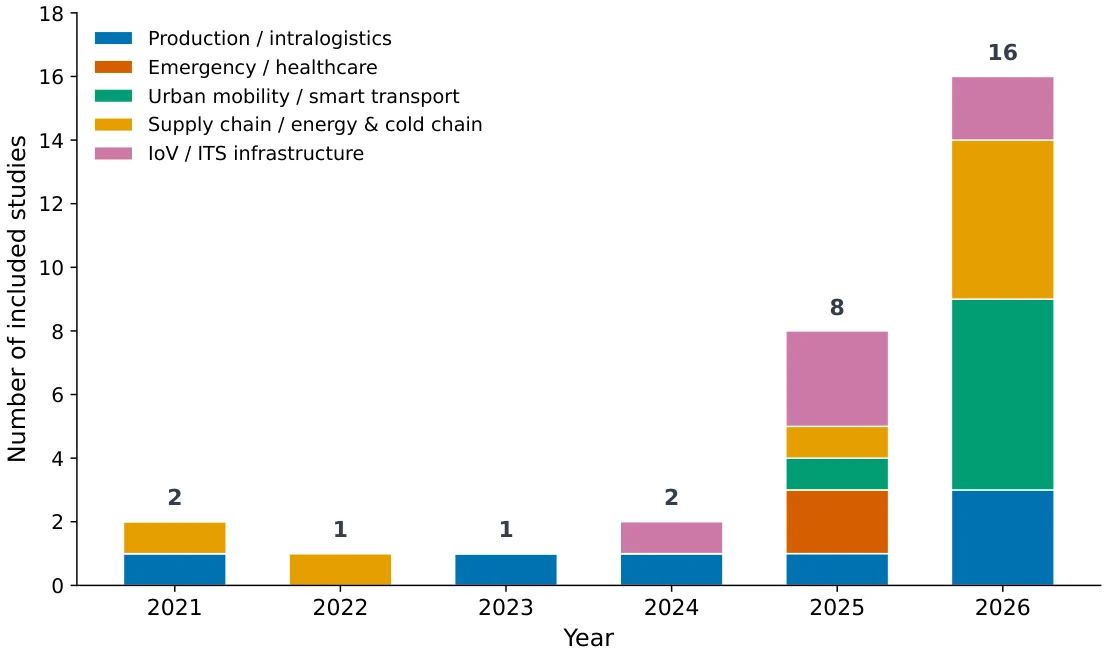
Included studies by year and application domain (stacked bars; the value above each bar is the annual total, n=30). The corpus is recent and concentrated in 2025–2026, and domain coverage broadens over time. The figure is descriptive of when and where research has appeared and should not be read as a statistical trend or forecast; the 2026 column is also the cohort captured by the Stage 2 update search (Section 3.4).

**Figure 3:**
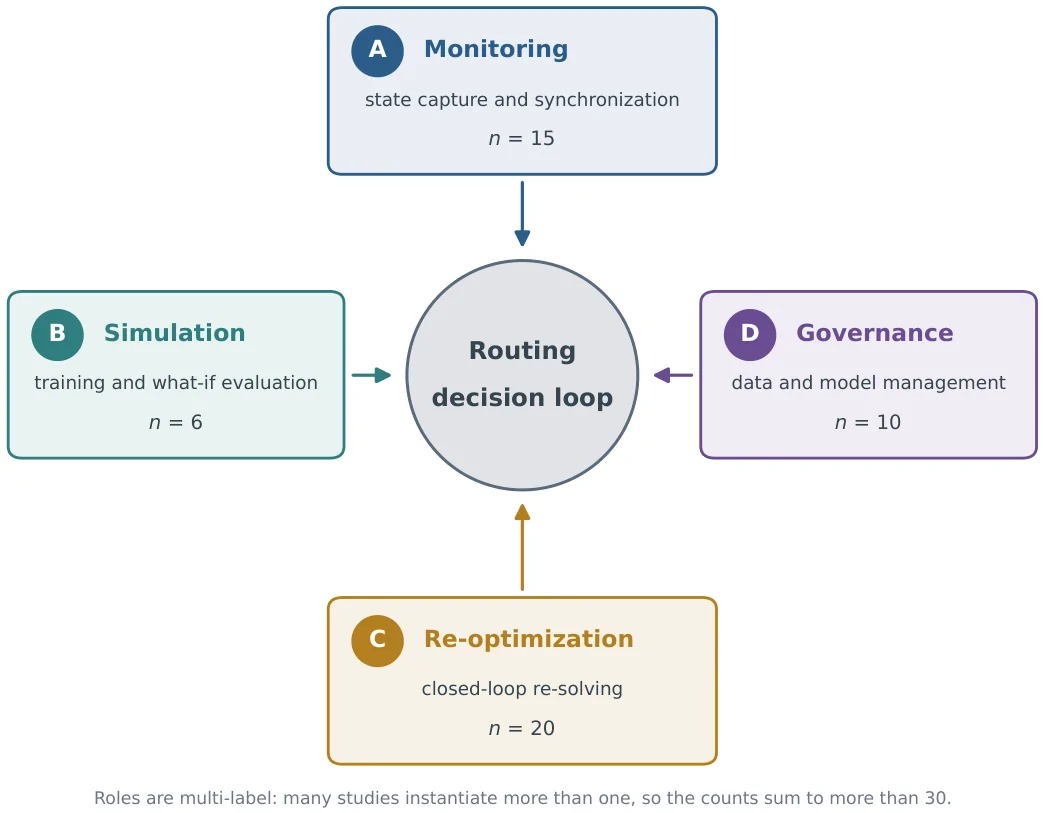
Taxonomy of the four roles a digital twin plays in the routing decision loop: (**A**), real-time monitoring and synchronization; (**B**), simulation and training; (**C**), adaptive decision-making and re-optimization; (**D**), infrastructure, data governance, and model management. Counts indicate how many of the 30 studies instantiate each role and sum to more than 30 because many studies fill several roles. The taxonomy shows that a digital twin’s contribution differs by role, which should be borne in mind when comparing studies. The four roles are an analytical classification defined a priori (Section 3.7), not a taxonomy induced from the corpus.

**Figure 4:**
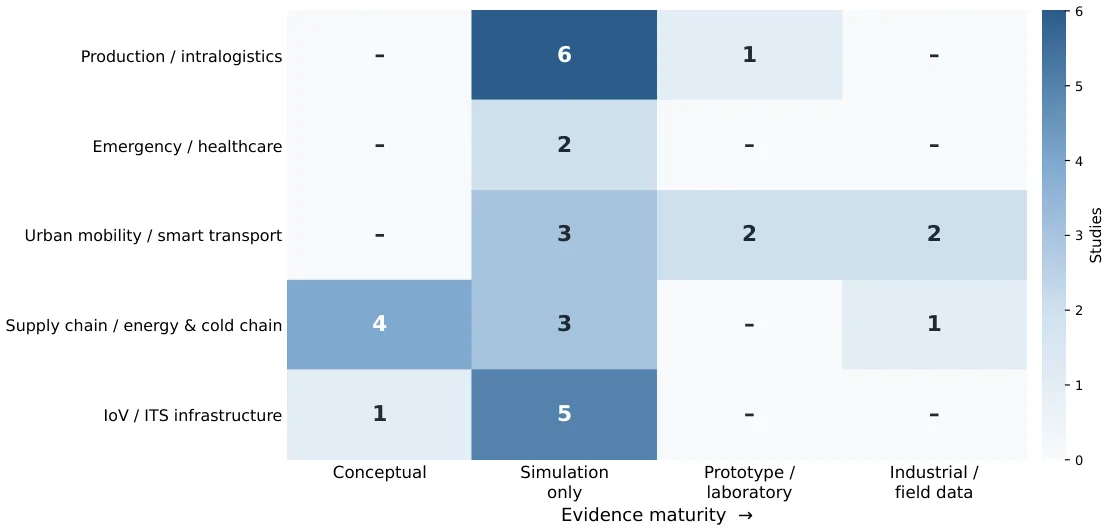
Distribution of the 30 included studies by application domain (rows) and evidence maturity (columns); each cell gives the number of studies and darker shading indicates higher counts. Validation is concentrated in the simulation-only column, with few prototype or field-validated studies, so reported performance benefits should be read as conditional on simulation evidence. Study identifiers are listed in Supplementary Materials Table S1.

**Figure 5:**
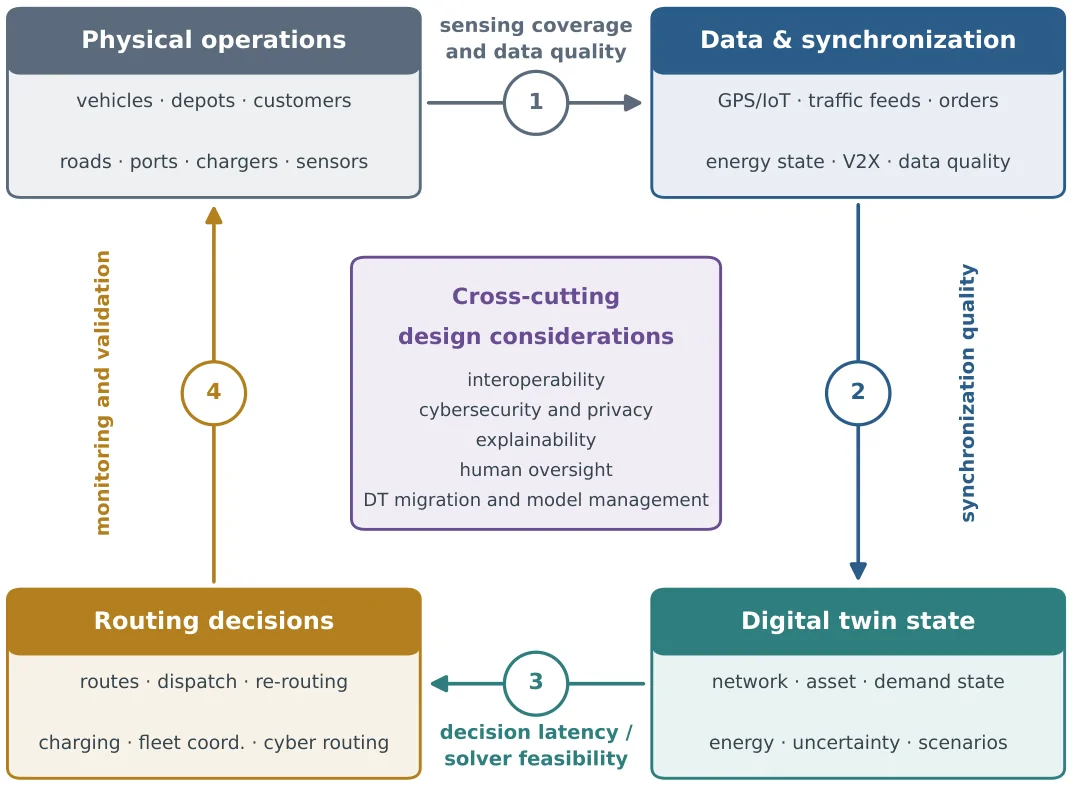
Routing-specific reference architecture shown as a closed control loop. The four numbered steps are the inter-stage constraints of the loop: (1) sensing coverage and data quality, from physical operations to data and synchronization; (2) synchronization quality, from data and synchronization to the digital-twin state; (3) decision latency and solver feasibility, from the twin state to routing decisions; and (4) monitoring and validation, the feedback path returning to physical operations. The center panel lists deployment-dependent cross-cutting design considerations; their relevance varies with architecture, governance setting, safety criticality, and whether the twin is co-located or distributed. The figure is not proposed as a new generic digital-twin architecture; it translates generic DT layers into a routing-specific control-loop model whose evidential basis, component by component, is given in Table 8.

**Figure 6:**
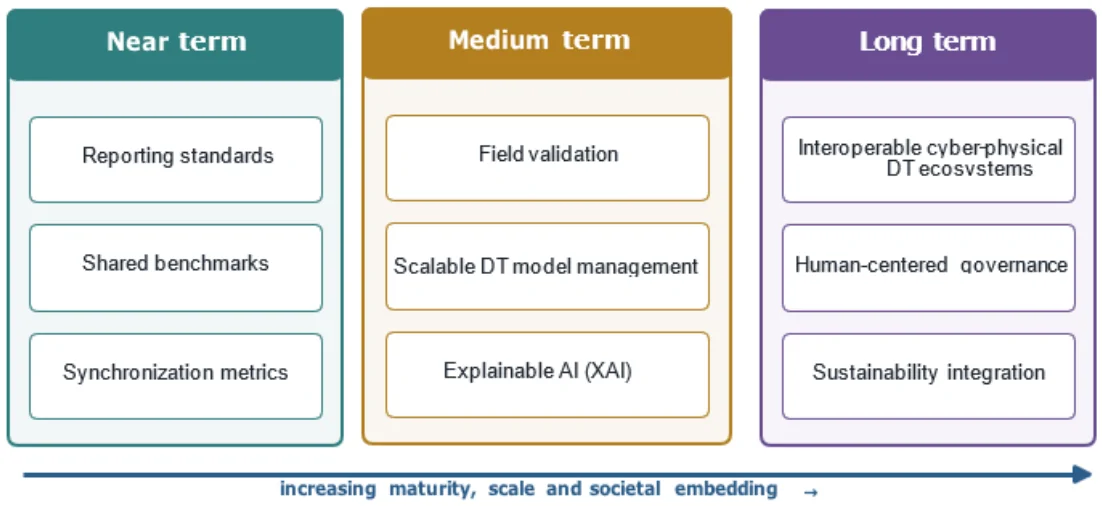
Research agenda across near-, medium-, and long-term horizons (three priorities per horizon). The rightward arrow indicates increasing maturity, scale, and societal embedding. The figure summarizes the agenda derived from the synthesis and the evidence-informed hypotheses (Section 7); the detailed rationale and evidential basis are given in the text and Table 9.

**Table 1:** Search strategy and Boolean keyword blocks, with search limits and available reporting details; database-specific executed search strings were not retained.

Element	Specification
Databases	Scopus; ScienceDirect (Elsevier); IEEE Xplore; Google Scholar (also used for citation chasing)
Fields searched	Title, abstract, and keywords (database-dependent)
Stage 1—coverage	2019–2025 (core search)
Stage 2—coverage	2026 (update search; see Section 3.4)
Digital-twin term block	“digital twin” OR “digital-twin” OR “DT-driven” OR “traffic digital twin” OR “digital supply chain twin”
Routing/optimization block	“vehicle routing problem” OR VRP OR “dynamic vehicle routing” OR DVRP OR “pickup and delivery” OR “AGV routing” OR “path planning” OR “emergency vehicle routing” OR “traffic optimization” OR “multi-hop routing” OR “Internet of Vehicles” OR IoV OR “vehicular edge computing” OR V2V OR “truck platooning”
Combination	Digital-twin block AND routing/optimization block (conceptual blocks shown; verbatim executed strings not retained)
Other methods	Backward and forward citation searching of included studies
Language and types	English; peer-reviewed journal articles, conference papers, and book chapters; non-peer-reviewed records were not counted in the corpus
Deduplication	DOI matching, title normalization, and author/year cross-checking before screening
Date last searched	Not recorded (see Section 10)

**Table 2:** Information sources: why each was used, and its coverage or reproducibility limitation.

Source	Role in This Review	Limitation Acknowledged
Scopus	Primary multidisciplinary bibliographic index; provides the broadest simultaneous coverage of engineering, transport, operations-research, and computer-science venues, and was the reference point for deduplication.	Selective journal indexing; conference coverage in computer science is less complete than in dedicated repositories.
ScienceDirect (Elsevier)	Publisher platform used to capture Elsevier engineering, transport, and logistics journals in full text, including advance-online articles not yet indexed elsewhere.	Not a bibliographic database equivalent to Scopus: coverage is confined to one publisher, so it cannot be treated as an independent index.
IEEE Xplore	Required for the IoV, V2X, ITS, and wireless/edge/cloud DT literature, which is dominated by IEEE journals and conference proceedings that general indexes cover unevenly.	Discipline-bounded; contributes little to operations-research and supply-chain venues.
Google Scholar	Supplementary discovery and backward/forward citation chasing across sources; used to detect records missed by the three indexes above.	Undisclosed ranking, unstable result sets, and no exportable search history, so searches are not reliably reproducible. Used as an auxiliary source only; no records are attributed to it in isolation.

**Table 3:** Eligibility criteria applied during study selection.

Dimension	Inclusion	Exclusion
Topic	Operational integration of a DT with a routing, path-planning, dispatching, charging-route, or multi-hop communication-routing decision	Routing/VRP without a DT; DT without any routing or path decision
DT substance	DT provides at least one of: real-time synchronization, simulation/training, adaptive re-optimization, or data/model management	DT mentioned only as background or future work, with no operationalization
DT level [28]	Digital shadow (automated one-way update) admitted only where the updated state informs a routing decision; digital twin (bidirectional or iterative coupling) admitted directly	Digital model (no automated data exchange) not admitted; a data or model platform not admitted unless it supports synchronization, twin updating, or routing decision logic
Routing layer	Physical routing of vehicles, goods, or responders; cyber-layer routing admitted only where it conditions the availability, latency, reliability, or synchronization of information used for a routing decision	Communication, offloading, or migration studies with no demonstrable bearing on a routing decision loop
Domain	Manufacturing/intralogistics, warehousing, emergency and healthcare logistics, smart-city mobility, cold chain, electric freight, IoV/vehicular edge	Out-of-scope domains with no routing relevance (e.g., DT for product design only)
Output	Empirical experiments, simulations, industrial/field datasets, optimization/learning algorithms, or system architectures	Editorials, abstract-only items, non-retrievable full texts
Source type	Peer-reviewed journal, conference, or book chapter	Non-peer-reviewed magazine/blog/white-paper or repository material (cited as background only)
Period	2019–2026	Pre-2019, except foundational VRP/DT works
Language	English	Non-English without available translation

**Table 4:** Evidence-confidence categories, the decision rule used to assign them, and their use in the synthesis.

Category	*n*	Definition	Assignment Rule	Use in the Synthesis
High	3	Industrial/field-data validation; clear DT operationalization and routing formulation; baseline comparison; multiple relevant metrics	All three inputs satisfied: validation on industrial or field data and clear DT operationalization and clear routing formulation	Supports stronger, field-relevant claims (e.g., [22,36,37])
Moderate	22	Simulation or prototype validation with clear methods and baselines but no field deployment	Simulation or prototype validation and both DT operationalization and routing formulation clearly reported	Supports mechanism plausibility and design guidance, not field-level effectiveness
Low	5	Conceptual architectures, reviews, or weak DT operationalization with limited validation	No validation of the authors’ own (conceptual or secondary review), or weak DT operationalization, or routing formulation not stated	Supports agenda-setting and framing only; never used to support a claim about operational performance
Background (not in corpus)	–	Non-peer-reviewed or non-routing sources (e.g., [25,26,38,39])	Fails the source-type or topic criterion in Table 3	Cited for context; not used as evidence of DT–VRP performance

**Table 5:** Cross-domain comparison of the 30 included studies. The final two columns are synthesis-level interpretations derived from the study-level coding in Supplementary Materials Table S1, not bibliographic fields extracted directly from the studies; Section 3.8 describes how they were derived, and the narrative interpretation is given in the text.

Domain	*n*	Dominant Routing Task	Dominant DT Role	Typical Validation	Main Evidence Strength (Interpretation)	Main Evidence Limitation (Interpretation)
Production and intralogistics	7	AGV routing and dispatch; VRP with pickup and delivery	A, C	Simulation (one prototype)	Clear DT–routing formulation; one prototype-validated study	Sim-to-real transfer largely unproven
Emergency and healthcare	2	Dynamic emergency-vehicle routing and rerouting	A, C	Simulation only	Direct response-time relevance	Very small evidence base; no field trials
Urban mobility	7	Traffic-aware routing; EV routing and charging; platooning	A, C	Conceptual to field	Hosts most field-grounded studies	Scale and data noise; heterogeneous metrics
Supply chain and energy	8	Cold-chain and energy-aware freight routing	C, D	Conceptual to field (energy)	Sustainability- and governance-aware routing	Several case-bounded; mixed maturity
IoV and infrastructure	6	Multi-hop and communication routing; DT migration	C, D	Conceptual to simulation	Twin reliability and placement treated as routing factors	Mostly simulation; enabling layer rather than physical VRP

**Table 6:** Solver/algorithm families across the corpus, with representative studies, typical validation, and typical reported metrics. Families are not mutually exclusive: studies combining method families are counted in each applicable family, so the counts sum to slightly more than 30. The table maps where each family has been applied; because domains, baselines, validation settings, and metrics differ across families, it does not support comparison of their effectiveness.

Solver/Algorithm Family	*n*	Representative Studies	Typical Validation	Typical Reported Metrics
Hybrid AI–optimization	9	[15,18,21,22,37]	Simulation → field	Cost, energy, travel time, quality, robustness
Reinforcement/deep RL	5	[13,16,24,42]	Simulation	Travel time, makespan, throughput, overhead
Metaheuristics/heuristics	4	[6,7,9,14]	Simulation	Distance, latency, response time
Multi-agent systems	2	[23,48]	Simulation	Latency, delivery ratio, load balance
MPC/control-based planning	2	[17,44]	Prototype/simulation	Congestion, tracking error, emissions
ML perception & analytics	3	[36,41,43]	Prototype/field	Detection accuracy, mobility KPIs
Exact optimization	1	[19]	Case study	Cost, computational performance
Conceptual/review/network analysis	6	[2,34,46,47]	Conceptual/architecture	Qualitative (no single metric)

**Table 7:** Proposed synchronization and data-freshness indicators for evaluating DT-enabled routing. These are evaluation variables proposed by this review, not measures reported by the included studies: only 2 of the 30 studies report an explicit synchronization measure and 8 report a proxy.

Indicator	Definition	What It Exposes
State staleness (Age of Information)	$\mathrm{AoI}=t_{\mathrm{decision}}-t_{\mathrm{last}\;\mathrm{update}}$, per state variable, at the moment a route is computed [49]	How old the state was when it was acted on; the single most direct measure of whether the twin was current
Update latency	Elapsed time from a physical event to the corresponding change in twin state	Where delay accumulates in the sensing and synchronization path
Decision latency	Elapsed time from twin-state update to an issued routing decision	Whether the solver is fast enough for the decision cycle it serves
State divergence	$∥x_{\mathrm{real}}\left(t\right)-x_{\mathrm{DT}}\left(t\right)∥$, or per-variable MAE/RMSE against ground truth	How far the twin drifts from reality, independently of how quickly it updates
Synchronization completeness	Proportion of the state variables a routing decision requires that were updated within the decision window	Partial synchronization, which staleness alone will not reveal
Communication reliability	Packet loss, missing-update rate, V2X link availability over the decision window	Whether the synchronization path itself is dependable in the deployment setting
Prediction drift	Growth of prediction error between successive twin updates	How long a prediction remains usable, which sets the required update rate
Route degradation under stale data	Change in travel time, cost, or service level when updates are artificially delayed	The quantity Hypothesis 1 requires: the routing consequence of poor synchronization

**Table 8:** Traceability from study-level coding to the reference-architecture components and design hypotheses. “Status” uses the four-level scale of Section 3.8: demonstrated (directly reported by multiple included studies); pattern (a cross-study regularity identified through the coding); inferred requirement (derived by the authors from a repeated gap or system dependency); hypothesis (proposed for future empirical testing).

Component or Hypothesis	Supporting Coding Dimension	Supporting Domains and Studies	Evidence Maturity	Status
Sensing and state capture (step 1)	DT role A; coupling granularity	All five domains; object to fleet scale [8,14,41,43]	Simulation to prototype	Demonstrated
Synchronization and model update (step 2)	DT role A; sync-metric field	Production, urban, IoV [6,24,41]	Simulation; 2 studies report an explicit measure	Pattern; measurement is an inferred requirement
Simulation and prediction	DT role B	Production, supply chain [13,16,22,42]	Simulation, one field	Demonstrated
Optimization and re-optimization (closed loop)	DT role C (n=20)	All five domains [9,15,18,21]	Simulation dominant	Demonstrated
Decision execution under latency (step 3)	Solver lens; performance-metric field	Urban, production [16,17,44]	Simulation to prototype; rarely measured	Inferred requirement (repeated gap)
Monitoring and field validation (step 4)	Evidence maturity	Urban, supply chain [22,36,37]	Field (n=3 of 30)	Inferred requirement (repeated gap)
DT distribution and migration	DT role D; cyber-layer coupling	IoV only [24,34,48]	Simulation and conceptual	Pattern; scope limited to distributed and edge–cloud deployments
Uncertainty as a routing-state variable	Coded routing task; solver lens	Supply chain, emergency [9,21]	Simulation	Inferred requirement (asymmetric coverage)
Communication state as a routing-state variable	Cyber-layer coupling	IoV [7,23,34]	Simulation and conceptual	Pattern; scope limited to connected deployments
Governance, security, interoperability	DT role D; checklist item 10	Supply chain, IoV [20,34,46]	Conceptual	Inferred requirement; deployment-conditional
Explainability and human oversight	Performance-metric field (absent)	Emergency, urban [9,14,44]	Not measured in any study	Inferred requirement (repeated absence)
Hypothesis 1 (synchronization and timeliness)	DT role A; sync-metric field; solver lens	Cross-domain [16,24,41]	Simulation; not directly tested	Hypothesis
Hypothesis 2 (model management and scalability)	DT role D; evidence maturity	IoV, supply chain, urban [22,24,37]	Simulation to field; no controlled comparison	Hypothesis (provisional support; not a study-level confidence rating)
Hypothesis 3 (co-modeled sustainability)	Coded routing task; performance-metric field	Supply chain, urban [18,21,22]	One field, two simulation	Hypothesis

**Table 9:** Research gaps, their evidential basis, and the corresponding future-research directions.

Research Gap (Evidential Basis)	Why It Matters	Research Direction	Horizon
Synchronization quality and data freshness rarely measured (2 of 30 studies report an explicit measure; Section 6.5; Hypothesis 1)	The defining DT property cannot be isolated from solver effects	Standardized benchmarks and reporting of the indicators in Table 7	Short
Simulation-only dominance; few field validations (Figure 4; Hypothesis 2)	Reported gains are largely in silico	Prototype and field-validation studies; staged deployment with model management	Short–medium
Explainability and trust essentially unmeasured (Section 8)	Opaque policies impede safety-critical adoption	The explainability roadmap of Section 9.1: policy explanation, reward decomposition, counterfactuals, audit trails, and quantitative trust metrics	Short–medium
Sustainability weakly coupled to routing (Section 5.4 and Section 7; Hypothesis 3)	Rhetorical sustainability does not change routes	Joint energy–infrastructure–emissions routing models	Medium
Limited supply-chain and reverse-logistics integration (Section 5.4)	Twins stop at the route, not the chain	End-to-end DT–VRP from procurement to reverse logistics	Medium
Interoperability and governance gaps; missing standards (Section 8)	Twins remain isolated islands	Open data models, interoperability standards, privacy/security, and governance frameworks	Medium–long
No study evaluates routing under adversarial or degraded communication (Section 9.2)	Corrupted twin updates corrupt every downstream routing decision	Routing evaluation under jamming, spoofing, and link loss; game-theoretic treatment of the synchronization path	Medium
Scalability of high-fidelity twins (Section 8)	Fidelity competes with real-time responsiveness	Adaptive-fidelity twins, edge–cloud model splitting (Section 9.2), and fine-grained model migration	Medium–long
Physical–cyber routing under-integrated (Section 5.5)	Physical routing increasingly depends on information flow	Unified twins jointly modeling goods movement and communication routing	Long

## Data Availability

No new primary data were generated. The data supporting this review are derived from the published studies cited in the article and from the study-level coding matrix provided as Supplementary Materials Table S1, which records the study-level classifications underlying the review’s principal descriptive results.

## Supplementary Materials

The following supporting information can be downloaded at: https://www.mdpi.com/article/10.3390/s26195986/s1, Table S1: Study-level coding matrix for the 30 included studies, including evidence type, routing layer, search stage, and synchronization reporting, together with the Stage 1/Stage 2 distribution comparison, reporting-completeness checklist, and coding legend.

## Abbreviations

The following abbreviations are used in this manuscript:

AGV	Automated guided vehicle
AoI	Age of Information
DRL	Deep reinforcement learning
DT	Digital twin
DVRP	Dynamic vehicle routing problem
EV	Electric vehicle
IoT	Internet of Things
IoV	Internet of Vehicles
ITS	Intelligent transportation systems
MPC	Model-predictive control
PRISMA	Preferred Reporting Items for Systematic Reviews and Meta-Analyses
PRISMA-S	PRISMA extension for reporting literature searches
RL	Reinforcement learning
V2V	Vehicle-to-vehicle
V2X	Vehicle-to-everything
VRP	Vehicle routing problem
VRPPD	Vehicle routing problem with pick-up and delivery

## References

[1] Abideen A.Z., Sundram V.P.K., Pyeman J., Othman A.K., Sorooshian S. (2021). Digital twin integrated reinforced learning in supply chain and logistics. Logistics.

[2] Vashishth T.K., Sharma V., Sharma K.K., Kumar B., Chaudhary S., Panwar R. (2025). Digital twins solutions for smart logistics and transportation. Digital Twins for Smart Cities and Villages.

[3] Dantzig G.B., Ramser J.H. (1959). The truck dispatching problem. Manag. Sci..

[4] Laporte G. (1992). The vehicle routing problem: An overview of exact and approximate algorithms. Eur. J. Oper. Res..

[5] Toth P., Vigo D. (2014). Vehicle Routing: Problems, Methods, and Applications.

[6] Pan Y.H., Wu N.Q., Qu T., Li P.Z., Zhang K., Guo H.F. (2021). Digital-twin-driven production logistics synchronization system for vehicle routing problems with pick-up and delivery in industrial park. Int. J. Comput. Integr. Manuf..

[7] Cong Dao T., Cong Luong N., Hung Nguyen N., Li X., Niyato D., Kim D.I. (2025). Multihop routing for IoT-based digital twin: Novel metaheuristic approaches. IEEE Internet Things J..

[8] Chen C.Y.T., Sun E.W., Lin Y.B. (2025). Reconciling spatiotemporal conjunction with digital twin for sequential travel time prediction and intelligent routing. Ann. Oper. Res..

[9] Abdelnabi A.B., Rabadi G. (2025). Real-time medical aid delivery: A digital twin approach with dynamic vehicle routing problem. Proceedings of the 2025 IEEE 5th International Conference on Digital Twins and Parallel Intelligence (DTPI).

[10] Grieves M., Vickers J. (2017). Digital twin: Mitigating unpredictable, undesirable emergent behavior in complex systems. Transdisciplinary Perspectives on Complex Systems.

[11] Tao F., Zhang H., Liu A., Nee A.Y.C. (2019). Digital twin in industry: State-of-the-art. IEEE Trans. Ind. Inform..

[12] Rasheed A., San O., Kvamsdal T. (2020). Digital twin: Values, challenges and enablers from a modeling perspective. IEEE Access.

[13] Zhang L., Yang C., Yan Y., Cai Z., Hu Y. (2024). Automated guided vehicle dispatching and routing integration via digital twin with deep reinforcement learning. J. Manuf. Syst..

[14] Ikram T., Sajjad M.A., Sajid J., Asayesh A., Malik A.W. (2025). Digital twin assisted real-time emergency vehicle navigation for resource optimization and reroute planning. Int. J. Parallel Emergent Distrib. Syst..

[15] Lou P., Zhong Y., Hu J., Fan C., Chen X. (2023). Digital-twin-driven AGV scheduling and routing in automated container terminals. Mathematics.

[16] Lee D., Kang Y.S., Noh S.D. (2026). Digital twin-driven deep reinforcement learning for real-time optimisation in dynamic AGV systems. Int. J. Prod. Res..

[17] Lis M., Mądziel M. (2026). Green transportation planning for smart cities: Digital twins and real-time traffic optimization in urban mobility networks. Appl. Sci..

[18] Xie Y., Li S., Lu L., Pan Y., Iida F. (2026). A digital twin-based approach for dynamic traffic-aware routing and charging of electric vehicles. Expert Syst. Appl..

[19] Marmolejo-Saucedo J.A. (2022). Digital twin framework for large-scale optimization problems in supply chains: A case of packing problem. Mob. Netw. Appl..

[20] Alam M.M., Nurkat Ugli R.A., Tanha K.J., Jun T. (2026). AI-enhanced digital twin systems for warehouse logistics optimization: A review of challenges with solutions and future directions. ICT Express.

[21] Nozari H., Yordanova Z. (2026). An adaptive fuzzy multi-objective digital twin framework for multi-depot cold-chain vehicle routing in agri-biotech supply networks. Logistics.

[22] Kaarlela T., Alekseev S., Maljamäki P., Ikäheimo E., Kurvinen E. (2026). Digital twin framework for electric truck route and infrastructure planning in the Nordic region. Transp. Eng..

[23] Alam M.Z., Khan K.S., Jamalipour A. (2024). Multiagent best routing in high-mobility digital-twin-driven Internet of Vehicles (IoV). IEEE Internet Things J..

[24] Xing L., Li B., Deng K., Gao J., Wu H., Ma H. (2026). Fine-grained model-level digital twin migration method for intelligent transportation systems. IEEE Open J. Intell. Transp. Syst..

[25] Nag D., Brandel-Tanis F., Pramestri Z.A., Pitera K., Frøyen Y.K. (2025). Exploring digital twins for transport planning: A review. Eur. Transp. Res. Rev..

[26] Li J., Wang J. (2025). Digital twin-driven management strategies for logistics transportation systems. Sci. Rep..

[27] Page M.J., McKenzie J.E., Bossuyt P.M., Boutron I., Hoffmann T.C., Mulrow C.D., Shamseer L., Tetzlaff J.M., Akl E.A., Brennan S.E. (2021). The PRISMA 2020 statement: An updated guideline for reporting systematic reviews. BMJ.

[28] Kritzinger W., Karner M., Traar G., Henjes J., Sihn W. (2018). Digital twin in manufacturing: A categorical literature review and classification. IFAC-PapersOnLine.

[29] Alavi H. (2022). Building Information Modeling for Facility Managers. Ph.D. Thesis.

[30] Alavi H., Kookalani S., Rahimian F.P., Forcada N. (2024). Introduction of methodology for BIM & DSS. Integrated Building Intelligence.

[31] Alavi H., Kookalani S., Rahimian F.P., Forcada N. (2024). Integrated Building Intelligence.

[32] Alavi H., Chen W., Wang M., Kookalani S., Brilakis I. Enhancing building maintenance efficiency through BIM–LiDAR integration. Proceedings of the 2024 European Conference on Computing in Construction (EC3 2024).

[33] Bortolini R., Rodrigues R., Alavi H., Dalla Vecchia L.F., Forcada N. (2022). Digital twins’ applications for building energy efficiency: A review. Energies.

[34] Zhang M., Guan K., He D., Hrovat A., Javornik T. (2026). Digital twin of wireless channels for the intelligent transportation system. IEEE Potentials.

[35] Rethlefsen M.L., Kirtley S., Waffenschmidt S., Ayala A.P., Moher D., Page M.J., Koffel J.B., PRISMA-S Group (2021). PRISMA-S: An extension to the PRISMA statement for reporting literature searches in systematic reviews. Syst. Rev..

[36] Rubio A., Marín Saltó A., Diaz F., Olivencia S. (2026). Vehicle Usage Digital Twins: Mobility Analysis Test Case.

[37] Guo X., Li Y., Cui S., Zhong R.Y. (2026). Digital twin-enabled cooperative autonomous truck platooning framework for modeling, cost estimation, and decision-making in transportation systems. Transp. Res. Part E Logist. Transp. Rev..

[38] Nonomura K., Wang K., Li Z., Yu T., Sakaguchi K. (2025). Digital twin-empowered cooperative autonomous car-sharing services: Proof-of-concept. arXiv.

[39] Ertoy Sarıışık B., Yalçıner Ercoşkun Ö. (2026). The role of digital twin technologies in resilient cities. Romaya J..

[40] Zhang B., Gao H., Sun P., Xu X., Wang L., Jia Y. (2025). Path planning for multi-autonomous vehicles based on digital twin technology. Digit. Twin.

[41] Li J., Song R., Liu H., Zhao E. (2026). The automatic packing box positioning of production line in digital twin logistics system based on YOLOv8. Digit. Twins Appl..

[42] Fu T., Zhu H. (2026). Digital-twin-based reinforcement learning method for flexible assembly job-shop scheduling with dynamic material kitting. Comput. Ind. Eng..

[43] Wang H., Xiao P., Liu A., Zhang J., Shan Q. (2026). UTDT: A baseline benchmark for urban traffic flow digital twins via roadside LiDAR–camera fusion. IEEE Trans. Intell. Transp. Syst..

[44] Yang H., Liu M., Guo G., Mu C. (2026). Digital twin-based trajectory planning and feedforward-feedback control for an autonomous vehicle on urban roads. IEEE Trans. Intell. Transp. Syst..

[45] Jiang H., Guo Z., Zhang Z. (2026). Digital twin and path planning for intelligent port inspection robots. J. Mar. Sci. Eng..

[46] Chen Q., Wang X., Liu L. (2026). Next-generation AI-driven digital twin framework for market volatility warning and risk detection in global logistics supply chains. IEEE Commun. Stand. Mag..

[47] Zhang H., Zheng Z., Li Y., Lu L. (2025). Digital twin-based analysis of V2V time-varying complex networks. IEEE Trans. Veh. Technol..

[48] Zahangir Alam M., Rahman S., Asif Bin Khaled M., Islam A., Jamalipour A. (2025). A graph-assisted digital-twin-driven multiagent shared offloading for Internet of Vehicles. IEEE Internet Things J..

[49] Kaul S., Yates R., Gruteser M. (2012). Real-time status: How often should one update?. Proceedings of the 2012 IEEE INFOCOM.

[50] Chen R., Yi C., Zhu H., Wu W., Kang J., Niyato D. (2026). Dynamic digital twin update by adaptive model splitting and reliable crowdsourcing under uncertain data distortions. IEEE Trans. Mob. Comput..

[51] Chen R., Yi C., Zhou F., Kang J., Wu Y., Niyato D. (2025). Federated digital twin construction via distributed sensing: A game-theoretic online optimization with overlapping coalitions. IEEE Trans. Mob. Comput..

[52] Chen R., Yi C., Zhu K., Chen B., Cai J., Guizani M. (2024). A three-party hierarchical game for physical layer security aware wireless communications with dynamic trilateral coalitions. IEEE Trans. Wirel. Commun..

